# Public perspectives on protective measures during the COVID-19 pandemic in the Netherlands, Germany and Italy: A survey study

**DOI:** 10.1371/journal.pone.0236917

**Published:** 2020-08-05

**Authors:** Karien Meier, Toivo Glatz, Mathijs C. Guijt, Marco Piccininni, Merel van der Meulen, Khaled Atmar, Anne-Tess C. Jolink, Tobias Kurth, Jessica L. Rohmann, Amir H. Zamanipoor Najafabadi, on behalf of the COVID-19 Survey Study group

**Affiliations:** 1 Leiden University Medical School, Leiden University, Leiden, The Netherlands; 2 Institute of Public Health, Charité—Universitätsmedizin Berlin, Berlin, Germany; Faculty of Science, Ain Shams University (ASU), EGYPT

## Abstract

**Background:**

The extent to which people implement government-issued protective measures is critical in preventing further spread of coronavirus disease 2019 (COVID-19) caused by coronavirus SARS-CoV-2. Our study aimed to describe the public belief in the effectiveness of protective measures, the reported implementation of these measures, and to identify communication channels used to acquire information on COVID-19 in European countries during the early stage of the pandemic.

**Methods and findings:**

An online survey available in multiple languages was disseminated starting on March 19th, 2020. After five days, we computed descriptive statistics for countries with more than 500 respondents. Each day, we assessed enacted community containment measures by stage of stringency (I-IV). In total, 9,796 adults responded, of whom 8,611 resided in the Netherlands (stage III), 604 in Germany (stage III), and 581 in Italy (stage IV). To explore possible dynamics as containment strategies intensified, we also included 1,365 responses submitted during the following week. Participants indicated support for governmental measures related to avoiding social gatherings, selective closure of public places, and hand hygiene and respiratory measures (range for all measures: 95.0%-99.7%). Respondents from the Netherlands less frequently considered a complete social lockdown effective (59.2%), compared to respondents in Germany (76.6%) or Italy (87.2%). Italian residents applied enforced social distancing measures more frequently (range: 90.2%-99.3%, German and Dutch residents: 67.5%-97.0%) and self-initiated hygienic and social distancing behaviors (range: 36.3%-96.6%, German and Dutch residents: 28.3%-95.7%). Respondents reported being sufficiently informed about the outbreak and behaviors to avoid infection (range: 90.2%-91.1%). Information channels most commonly reported included television newspapers, official health websites, and social media. One week later, we observed no major differences in submitted responses.

**Conclusions:**

During the early stage of the COVID-19 pandemic, belief in the effectiveness of protective measures among survey respondents from three European countries was high and participants reported feeling sufficiently informed. In March 2020, implementation of measures differed between countries and were highest among respondents from Italy, who were subjected to the most stringent lockdown measures and greatest COVID-19 burden in Europe during this period.

## Introduction

The recent pandemic of COVID-19 (coronavirus disease 2019) caused by SARS-CoV-2 (Severe Acute Respiratory Syndrome Coronavirus 2) has infected more than 9,200,000 people worldwide and caused more than 470,000 deaths as of June 24th, 2020 [1]. At that time, more than 1,500,000 COVID-19 cases and 175,000 deaths have been reported in the EU/EEA countries and UK combined [2]. This rapidly spreading virus imposes a tremendous burden on national healthcare systems as they lack sufficient material and human resources to respond to the sharply increasing number of patients requiring intensive care [1,3]. Worldwide, public health organizations as well as national and international government bodies have suggested systematic implementation of protective, public health measures in an effort to delay the spread of COVID-19 [4]. The aim of these measures is to decrease the peak infection rate, while maintaining a high quality of care under finite resources and limited hospital capacities [3,5]. In addition to basic hygienic advice, such as regular hand washing, the most important recommendation known to limit and delay the spread of the virus is social (physical) distancing [6,7].

In early March 2020, Europe became the epicenter of the COVID-19 pandemic, with more cases and deaths reported than in all other countries (excluding China) combined [1]. Throughout the course of March, most European countries progressively implemented community isolation measures to increase social distancing, such as imposing work restrictions and the closure of public places. Italy, the most severely affected European country in the early phase, imposed strict measures on March 9th and 11th, 2020. This included enforcing a nationwide quarantine in response to the alarming increase in the number of cases, which posed a serious threat to the capacity of the Italian healthcare system [8].

The aim of our study was to describe public belief in the effectiveness of protective measures, to what extent individuals have implemented these measures, and to identify key communication channels used to acquire information on COVID-19 in European countries. We believe these insights are not only valuable for the ongoing mitigation of the current pandemic, but may also serve to inform governments’ and public health organizations’ information dissemination and infection control strategies in the future.

## Materials and methods

### Design, setting, and participants

The survey instrument used to gather cross-sectional data was compiled by a team of medical students and researchers from the Leiden University Medical Center and the Charité—Universitätsmedizin Berlin. Our initial aim was to collect data on adults living in Europe, with an emphasis on individuals residing in the Netherlands, Germany, and Italy; however, the survey was also open to residents of other countries. We plan to continue data collection as community isolation measures remain in place. In these primary descriptive analyses, we only analyzed data from countries with at least 500 responses at our first cut-off date, March 23rd, 2020, which is in line with previous comparable studies [9–11]. This date was chosen because several European regional and national governing bodies announced stricter measures around this date.

The study was reviewed and granted exempted status from medical ethical approval by the Institutional Review Board of Leiden University Medical Center in The Netherlands (protocol number: N20-037).

### Survey instrument

We selected questions from the validated Flu TElephone Survey Template (FluTEST), which was designed to assess perceptions and behavior during an influenza pandemic [12]. We slightly modified the items to fit the current outbreak context and formulated additional questions to assess beliefs in the effectiveness of specific protective measures [13]. In brief, the survey instrument consisted of 22 total items in three sections: 1) five questions regarding beliefs in the effectiveness of public measures to reduce outspread (e.g. selective closure of places and complete social lockdown), 2) 16 questions on the personal application of protective measures (e.g. social distancing behaviors and hygienic practices), and 3) one question on the three most frequently used sources to acquire information about the outbreak and one question assessing whether or not respondents felt sufficiently informed. The full survey is presented in S1 Appendix. Additional questions captured sociodemographic information including gender, age, household composition, employment status, educational level, country of residence, being a healthcare provider or (bio)medical student, and prevalent chronic medical conditions. Additionally, respondents were asked to indicate the channel from which they were referred to the survey.

Survey data was collected and stored confidentially online using the database management system Qualtrics (Version March 2020, Qualtrics, Provo, UT, USA). On the first page of the survey, participants were requested to provide informed consent before they could proceed to respond to the items. Participants were not offered any financial incentive to participate in this short survey. The questionnaire could only be submitted once per device in an effort to reduce potential repeat responses.

The survey was translated from the original English version into multiple languages by native speakers, after which it was checked by at least two other native speakers. Then, the survey was shared with a small panel of native speakers, who provided us with further feedback on the understandability of the translation, before public dissemination. No back-translation was performed due to time constraints and the urgency to disseminate the survey. The survey went live on March 19th, 2020 in Dutch, English and German. Additional languages were added since initiation (Italian on March 20, 2020; French and Polish on March 21, 2020; Spanish on March 22, 2020; Turkish on March 25, 2020; and Farsi on March 29, 2020; see S1 Appendix).

### Procedures

The full survey was initially piloted on a sample of 50 respondents. After minor modifications to the structure and language, the survey was actively disseminated through (social) media channels, such as WhatsApp, Telegram, Facebook, LinkedIn, Instagram, and Twitter, and in professional networks via electronic mailing lists. The survey was further promoted via some local and national news websites and radio stations. On the landing page, participants were briefed about the study and only those providing informed consent for participation were guided to the 5-minute survey. On the final page, participants were debriefed about the study and thanked for their contribution.

### Assessment of stages of community containment measures

We compiled government-enacted community containment measures in each included country from public authority announcements and news articles from March 1st, 2020 until March 31st, 2020. Two independent researchers classified the stringency of isolation measures for each country in four stages based on the Community Containment Measures guideline developed by the Centers for Disease Control and Prevention (CDC) during the SARS outbreak in 2003 [14]. Any disagreement in staging was resolved by discussion. The CDC guideline describes seven interventions, which we grouped into four stages to create country-specific timelines. Guideline interventions 1 (passive monitoring), 2 and 3 (active monitoring without and with activity restrictions, respectively) were grouped together as Stage I (“Low Impact Containment Measures”), since most countries had already implemented these interventions in early March 2020. Guideline interventions 4 (working quarantine) and 5 (focused measures) were grouped and classified as Stage II (“Focused Measures to Increase Social Distance”), as many countries applied these interventions simultaneously. We designated intervention 6 as Stage III (“Community-Wide Measures to Increase Social Distance”) and intervention 7 as Stage IV (“Widespread Community Quarantine, Including Cordon Sanitaire”). We detailed the daily stage classification in country-specific timelines (S2 Appendix).

### Statistical analyses

Data were collected over a five-day period between March 19th, 2020 and March 23th, 2020 at 11:20 AM (UTC+0) for the primary analyses. We present results of the survey items including sociodemographic characteristics using descriptive summary statistics for the countries having more than 500 responses during this primary data collection period (the Netherlands, Germany, and Italy). Nominal variables were described and visualized using frequencies and percentages. We also reported frequencies of missing responses. We present stratified results for the assessed sociodemographic variables only for the Netherlands, as the number of responses was sufficient per individual subgroup. No formal statistical comparisons were made between countries since the primary aim was descriptive and there were no *a priori* testable hypotheses.

For the secondary analysis, we collected data for seven days immediately following the primary data collection period (through March 30th, 2020 at 11.40 AM (UTC+0)). As a secondary analysis, we explored changes in responses for items about the beliefs in the effectiveness of these measures and their implementation over time. As for items about implementation of protective measures, we reported the proportion of positive answers (“Yes”) out of all responses, excluding responses indicating the question was not applicable to their situation. Similarly, for items about the belief in the effectiveness of these measures, we considered the proportion of positive answers (“Probably true”). To visualize this, we modeled the proportions for each item and for each country separately, using generalized additive models with time as the independent variable, using a penalized cubic regression spline with three knots. In addition, we computed and presented visualizations of the differences in proportion between the responses recorded during the primary data collection period and the weeklong extension only for the Netherlands.

Data management, analyses and visualizations were conducted using Stata 16.1 (StatCorp LP, College Station, TX) software and R 3.6.3 / RStudio 1.2 [15,16].

## Results

**Fig 1:**
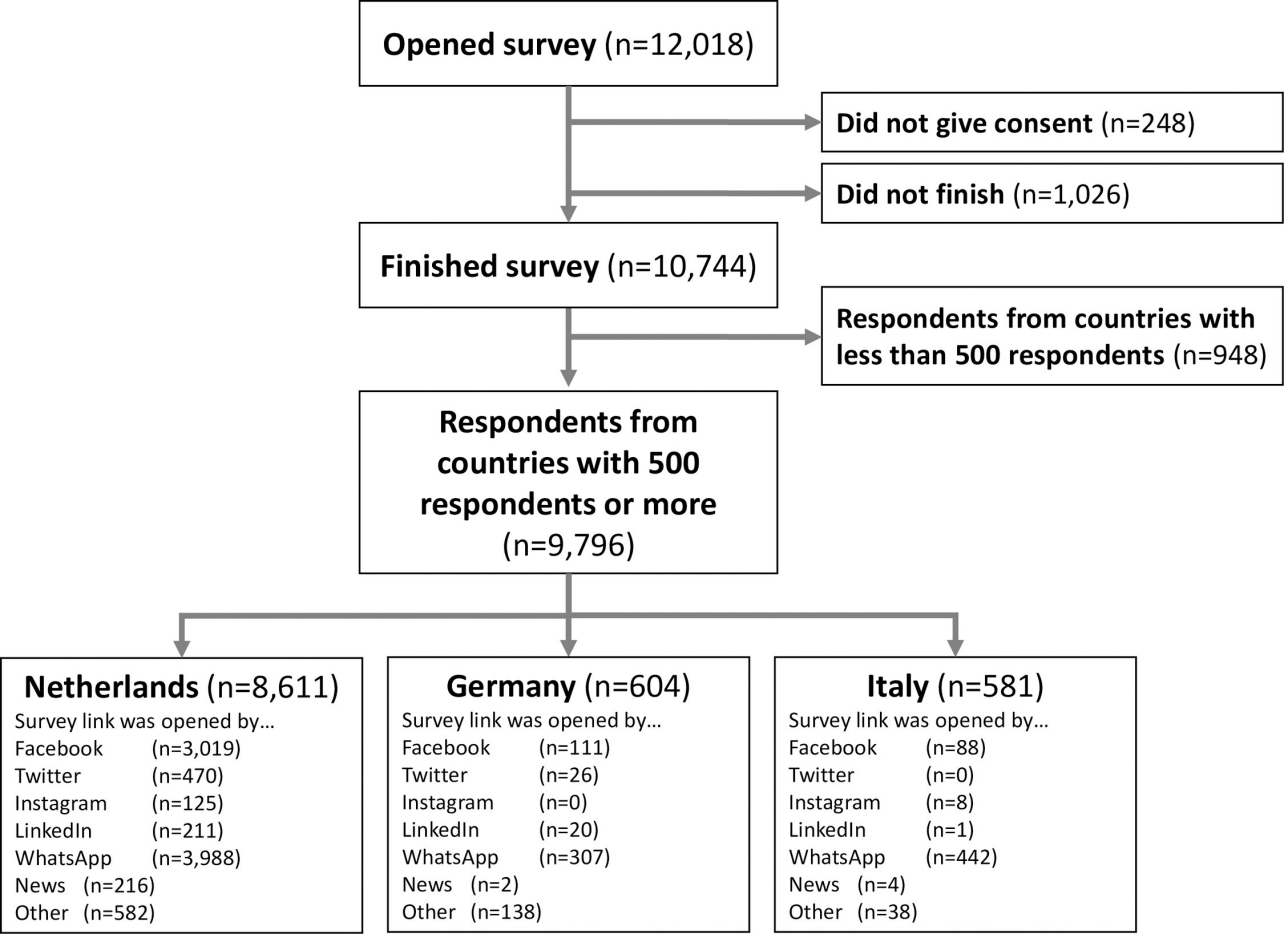
Flow chart of respondents in survey, on March 23rd, 2020.

Between March 19th and 23rd, 2020, a total of 9,796 respondents completed the survey. Three countries met our study inclusion criteria of having more than 500 respondents; the Netherlands (n = 8,611), Germany (n = 604), and Italy (n = 581) (see flowchart, Fig 1). The majority in all three countries opened the survey link by WhatsApp (range 46.3%-76.1%) or Facebook (range: 15.1%-35.1%). During this primary data collection period, the containment measures in the Netherlands and Germany met criteria for Stage III classification (“Community-Wide Measures to Increase Social Distance”), and those in Italy met Stage IV criteria (“Widespread Community Quarantine”).

Approximately two-thirds of respondents were female and one-third of respondents were aged 21–30 years old (Table 1). The majority of respondents had a paid job (57.1%) and many had tertiary academic degrees (68.2%). Approximately 18% of respondents were healthcare providers or (bio)medical students. Less than one-fifth of respondents reported suffering from a chronic illness or being in poor medical condition (17.3%). Descriptive sociodemographic characteristics stratified by country of residence are presented in Table 1.

**Table 1:** Sociodemographic characteristics of respondents on March 23rd, 2020, by country.

	Netherlands (Stage III)	Germany (Stage III)	Italy (Stage IV)	Total
No.	8,611	604	581	9,796
**Gender (%)**				
Male	2,492 (28.9)	206 (34.1)	192 (33.1)	2,890 (29.5)
Female	6,095 (70.8)	389 (64.4)	388 (66.8)	6,872 (70.2)
Other	24 (0.3)	9 (1.5)	1 (0.2)	34 (0.4)
**Age, years (%)**				
≤20 years	675 (7.8)	11 (1.8)	117 (20.1)	803 (8.2)
21–30 years	2,867 (33.3)	222 (36.8)	189 (32.5)	3,278 (33.5)
31–40 years	1,167 (13.6)	223 (36.9)	99 (17.0)	1,489 (15.2)
41–50 years	1,278 (14.8)	70 (11.6)	75 (12.9)	1,423 (14.5)
51–60 years	1,558 (18.2)	47 (7.8)	73 (12.6)	1,678 (17.1)
61–70 years	852 (9.9)	23 (3.8)	26 (4.5)	901 (9.2)
> 70 years	214 (2.5)	8 (1.3)	2 (0.3)	224 (2.3)
**Daily activity (%)**				
Paid job	4,885 (56.7)	388 (64.2)	321 (55.3)	5,594 (57.1)
Homemaker/Unemployed	573 (6.7)	41 (6.8)	37 (6.4)	651 (6.7)
Student	2,272 (26.4)	131 (21.7)	199 (34.3)	2,602 (26.6)
Retired	557 (6.5)	20 (3.3)	12 (2.1)	589 (6.0)
Other	324 (3.8)	24 (4.0)	12 (2.1)	360 (3.7)
**Household composition (%)**				
Single	1,391 (16.2)	113 (18.7)	94 (16.2)	1,598 (16.3)
Parent(s) with child(ren)	2,740 (31.8)	174 (28.8)	135 (23.2)	3,049 (31.1)
Living with partner	2,339 (27.2)	189 (31.3)	94 (16.2)	2,622 (26.8)
Shared flat (with roommates)	1,558 (18.1)	112 (18.5)	34 (5.9)	1,704 (17.4)
Other	583 (6.8)	16 (2.7)	224 (38.6)	823 (8.4)
**Highest educational qualification (%)**				
Primary/Secondary	635 (7.4)	132 (21.9)	301 (51.8)*	1068 (10.9)
Tertiary vocational	1,991 (23.1)	57 (9.4)		2048 (20.9)
Tertiary academic	5,985 (69.5)	415 (68.7)	280 (48.2)	6,680 (68.2)
**Healthcare provider/(bio-)medical student (%)**	1,572 (18.3)	123 (20.4)	48 (8.3)	1,743 (17.8)
**Chronic illness or being in poor medical condition (%)**	1,528 (17.7)	103 (17.1)	64 (11.0)	1,695 (17.3)
**Sources used to acquire information on COVID-19 (%)**				
Television	6,613 (76.8)	320 (53.0)	476 (82.0)	7,409 (75.6)
Newspaper, mobile news application	5,422 (63.0)	295 (48.8)	180 (31.0)	5,897 (60.2)
Social media	3,441 (40.0)	277 (45.9)	324 (55.8)	4,042 (41.3)
Radio	1,077 (12.5)	161 (26.7)	31 (5.3)	1,269 (13.0)
Official health hotlines	127 (1.5)	7 (1.2)	9 (1.6)	143 (1.5)
Official health websites	3,361 (39.0)	327 (54.1)	268 (46.1)	3,956 (40.4)
Healthcare professionals	381 (4.4)	26 (4.3)	44 (7.6)	451 (4.6)
People I speak to on a daily basis	2,293 (26.6)	161 (26.7)	139 (23.9)	2,593 (26.5)

For full questionnaire and wording, see S1 Appendix.

* Based on differences in the Italian education system, we considered primary, lower secondary school and upper secondary school as “Primary/Secondary” and university degrees as “Tertiary academic”.

### Sources used to acquire information on the COVID-19 outbreak

Among respondents living in the Netherlands, Germany, or Italy, the most frequently used sources to obtain relevant information included television (e.g. televised news, range: 53.0%-82.0%), newspapers or news applications (range: 31.0%-63.0%), social media (e.g. Facebook and Twitter, range: 40.0%-55.8%), and official health websites (range: 39.0%-54.1%). Other people (e.g. family, friends and colleagues, range: 23.9%-26.7%) and radio (range: 5.3%-26.7%) were reported less frequently. In all three countries, healthcare professionals (range: 4.3%-7.6%) and official health hotlines (range: 1.2%-1.6%) were the least frequently reported sources of information (Table 1). Almost all respondents living in these three countries reported feeling sufficiently informed about the current COVID-19 outbreak and what they could do to prevent an infection (range: 90.2%-91.1%; Table 2).

**Table 2:** Being informed about and belief in the effectiveness of policy recommendations during the early phase of the COVID-19 pandemic on March 23rd, 2020, by country.

		Netherlands (Stage III)	Germany (Stage III)	Italy (Stage IV)
No.		8,611	604	581
**Have been sufficiently informed (%)**	Probably true	7,839 (91.0)	545 (90.2)	529 (91.1)
	Probably false	271 (3.2)	23 (3.8)	29 (5.0)
	Not sure	476 (5.5)	33 (5.5)	21 (3.6)
	No opinion	25 (0.3)	3 (0.5)	2 (0.3)
**Belief in effectiveness of recommendations (%)**				
Avoid social gatherings	Probably true	8,479 (98.9)	594 (98.5)	576 (99.7)
	Probably false	60 (0.7)	4 (0.7)	2 (0.4)
	Don’t know	31 (0.4)	5 (0.8)	0 (0.0)
	Missing	41	1	3
Selective closure of public places/locations	Probably true	8,158 (95.3)	586 (97.2)	568 (98.1)
	Probably false	234 (2.7)	13 (2.2)	11 (1.9)
	Don’t know	172 (2.0)	4 (0.7)	0 (0.0)
	Missing	47	1	2
Implementation of hand hygiene measures	Probably true	8,240 (96.0)	589 (98.2)	567 (98.1)
	Probably false	169 (2.0)	4 (0.7)	8 (1.4)
	Don’t know	169 (2.0)	7 (1.2)	3 (0.5)
	Missing	33	4	3
Implementation of respiratory measures	Probably true	8,139 (95.0)	583 (97.3)	569 (99.0)
	Probably false	230 (2.7)	9 (1.5)	4 (0.7)
	Don’t know	196 (2.3)	7 (1.2)	2 (0.4)
	Missing	46	5	6
Complete social lockdown/isolation	Probably true	5,063 (59.2)	458 (76.6)	495 (87.2)
	Probably false	1,983 (23.2)	72 (12.0)	29 (5.1)
	Don’t know	1,500 (17.6)	68 (11.4)	44 (7.8)
	Missing	65	6	13

For full questionnaire and wording, see S1 Appendix.

Response percentages may not add up to 100% due to rounding.

### Belief in the effectiveness of measures to reduce outspread

**Fig 2:**
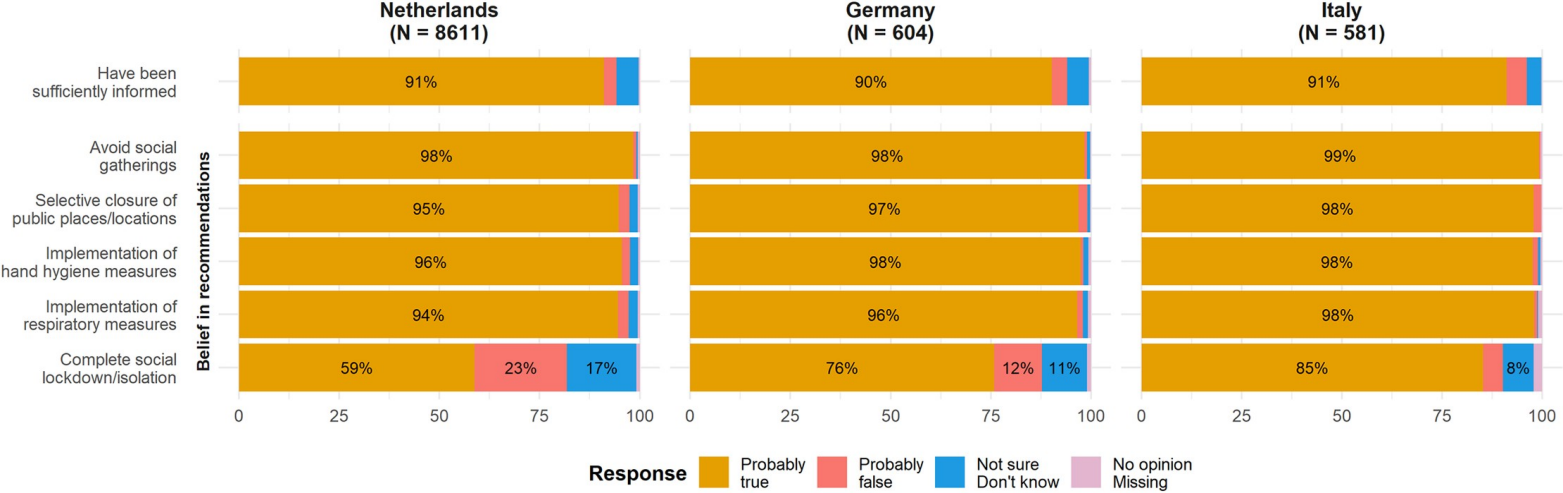
Being informed about and belief in the effectiveness of policy recommendations during the early phase of the COVID-19 pandemic on March 23rd, 2020, by country. Response percentages are rounded and may not add up to 100%. Percentages below 5% were omitted in the visualization.

The majority of respondents believed that avoiding social gatherings, the selective closure of public places and locations, hand hygiene measures, and respiratory measures were effective ways to prevent further spread of COVID-19 (range for all measures in all three countries: 95.0%-99.7%; Table 2). During this early pandemic phase, only 59.2% of respondents in the Netherlands perceived a complete social lockdown or isolation measures as effective, compared with 76.6% of respondents from Germany and 87.2% from Italy (Fig 2 and Table 2).

### Individual implementation of protective measures

**Fig 3:**
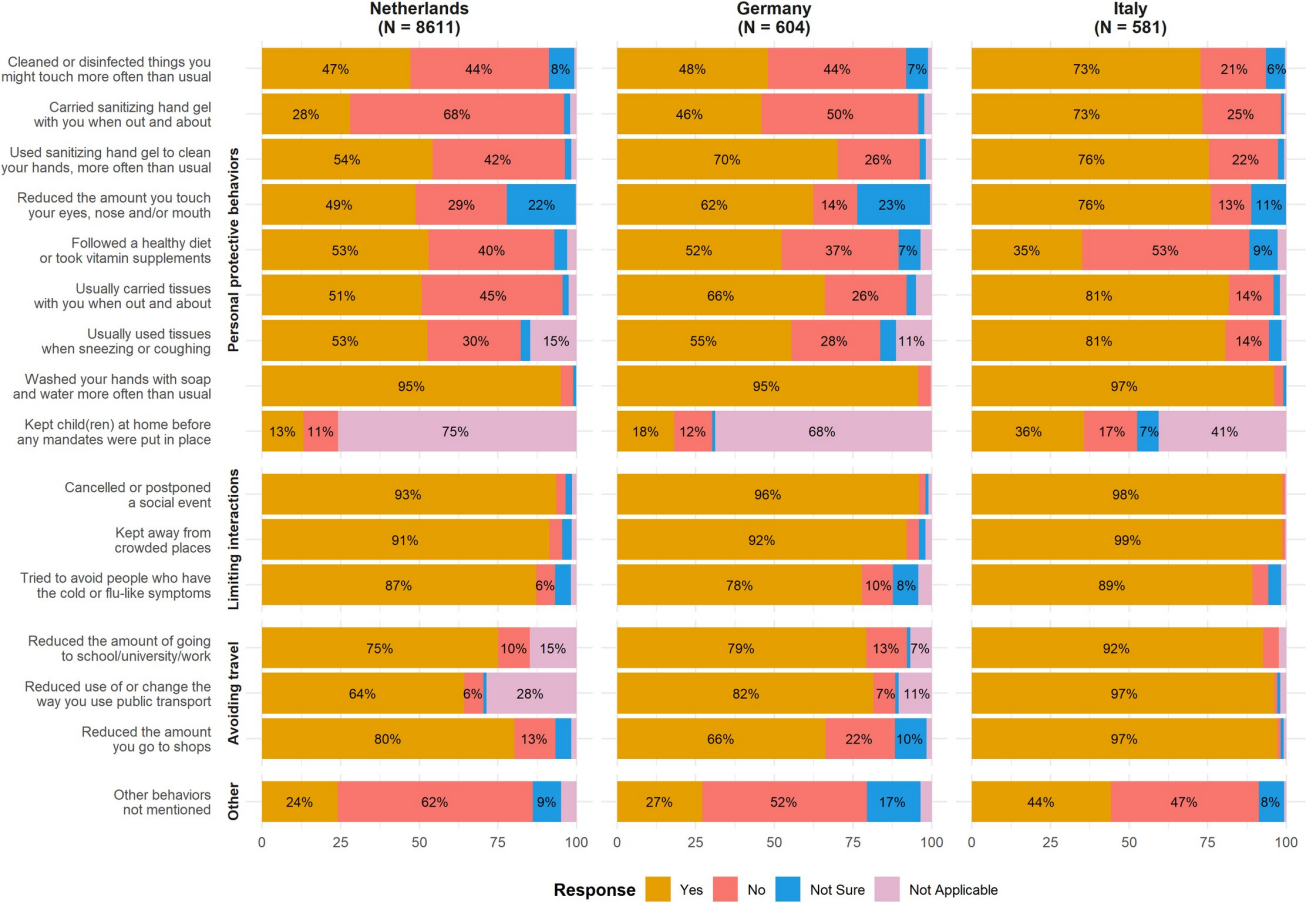
Individual implementation of protective measures in response to the COVID-19 pandemic (early phase) on March 23rd, 2020, by country. Response percentages are rounded and may not add up to 100%. Percentages below 5% were omitted in the visualization.

For all items, the percentages reported in the text and Table 3 excluded respondents to whom the item did not apply, which was especially important in the interpretation of three items (voluntarily keeping children at home before any mandates were put in place, range: 41.0%-75.1%; reducing the use of public transport, range: 1.9%-28.5%; and going to school/university/work, range: 2.4%-14.9%). With regard to personal protective behaviors, a high number of respondents from the Netherlands and Germany reported to have washed their hands with soap and water more often than usual (range: 95.0%-95.7%). In general, respondents from Italy reported applying all proposed personal protective behaviors more often than those from the Netherlands or Germany, except for following a healthy diet or using vitamin supplements (36.3%, Netherlands 54.5%, Germany 54.4%) (Fig 3 and Table 3).

**Table 3:** Individual implementation of protective measures in response to COVID-19 pandemic on March 23rd, 2020, by country.

	Netherlands (Stage III)				Germany (Stage III)				Italy (Stage IV)			
No.	8,611				604				581			
**Personal protective behaviors (%)**	**Y**	**N**	**NS**	**NA**	**Y**	**N**	**NS**	**NA**	**Y**	**N**	**NS**	**NA**
Cleaned or disinfected things you might touch more often than usual	4,083 (47.8)	3,753 (43.9)	713 (8.3)	62	292 (48.9)	264 (44.2)	41 (6.9)	7	423 (73.2)	123 (21.3)	32 (5.5)	3
Carried sanitizing hand gel with you when out and about	2,385 (28.3)	5,871 (69.6)	181 (2.2)	174	277 (47.0)	302 (51.2)	11 (1.9)	14	424 (73.5)	147 (25.5)	6 (1.0)	4
Used sanitizing hand gel to clean your hands, more often than usual	4,681 (55.3)	3,601 (42.5)	186 (2.2)	143	424 (71.5)	157 (26.5)	12 (2.0)	11	442 (76.6)	125 (21.7)	10 (1.7)	4
Reduced the amount you touch your eyes, nose and/or mouth	4,208 (49.0)	2,491 (29.0)	1,889 (22.0)	23	374 (62.2)	87 (14.5)	140 (23.3)	3	439 (75.7)	77 (13.3)	64 (11.0)	1
Followed a healthy diet or took vitamin supplements	4,548 (54.5)	3,429 (41.1)	370 (4.4)	264	317 (54.4)	226 (38.8)	40 (6.9)	21	205 (36.3)	308 (54.5)	52 (9.2)	16
Usually carried tissues with you when out and about	4,399 (52.4)	3,833 (45.7)	163 (1.9)	216	400 (69.7)	157 (27.4)	17 (3.0)	30	472 (83.0)	83 (14.6)	14 (2.5)	12
Usually used tissues when sneezing or coughing	4,542 (62.0)	2,551 (34.8)	232 (3.2)	1,286	335 (62.5)	169 (31.5)	32 (6.0)	68	468 (81.8)	82 (14.3)	22 (3.9)	9
Washed your hands with soap and water more often than usual	8,176 (95.0)	371 (4.3)	56 (0.7)	8	576 (95.7)	23 (3.8)	3 (0.5)	2	561 (96.6)	17 (2.9)	3 (0.5)	0
Kept child(ren) at home before any mandates were put in place	1,151 (53.6)	957 (44.6)	38 (1.8)	6,465	111 (58.1)	75 (39.3)	5 (2.6)	413	208 (60.6)	97 (28.3)	38 (11.1)	238
**Limiting interactions with people (%)**	**Y**	**N**	**NS**	**NA**	**Y**	**N**	**NS**	**NA**	**Y**	**N**	**NS**	**NA**
Cancelled or postponed a social event	8,042 (94.8)	268 (3.2)	178 (2.1)	123	580 (97.0)	11 (1.8)	7 (1.2)	6	572 (98.8)	6 (1.0)	1 (0.2)	2
Kept away from crowded places	7,834 (92.4)	367 (4.3)	277 (3.3)	133	555 (93.8)	22 (3.7)	15 (2.5)	12	575 (99.3)	3 (0.5)	1 (0.2)	2
Tried to avoid people who have the cold or flu-like symptoms	7,523 (89.0)	514 (6.1)	414 (4.9)	160	469 (81.1)	63 (10.9)	46 (8.0)	26	515 (90.2)	31 (5.4)	25 (4.4)	10
**Avoiding travel (%)**	**Y**	**N**	**NS**	**NA**	**Y**	**N**	**NS**	**NA**	**Y**	**N**	**NS**	**NA**
Reduced the amount of going to school/university/work	6,446 (88.0)	839 (11.5)	43 (0.6)	1,283	478 (84.9)	81 (14.4)	4 (0.7)	41	535 (94.4)	31 (5.5)	1 (0.2)	14
Reduced use of or change the way you use public transport	5,521 (89.6)	511 (8.3)	127 (2.1)	2,452	493 (91.3)	41 (7.6)	6 (1.1)	64	562 (98.6)	5 (0.9)	3 (0.5)	11
Reduced the amount you go to shops	6,888 (81.4)	1,154 (13.6)	421 (5.0)	148	401 (67.5)	134 (22.6)	59 (9.9)	10	563 (97.7)	8 (1.4)	5 (0.9)	5
Other behaviors not mentioned	2,094 (25.6)	5,335 (65.2)	760 (9.3)	422	166 (28.5)	316 (54.2)	101 (17.3)	21	254 (44.0)	274 (47.5)	49 (8.5)	4

Y = Yes; N = No; NS = Not sure; NA = Not applicable. For full questionnaire and wording, see S1 Appendix.

Response percentages may not add up to 100% due to rounding.

Behavior related to limiting interactions with people was fairly similar between countries. However, respondents from Italy more frequently reported cancelling or postponing social events (98.8%, compared with 94.8% in the Netherlands and 97.0% in Germany) and avoiding crowded places (99.3%, compared with 92.4% in the Netherlands and 93.8% in Germany). Respondents living in Germany less frequently reported avoiding people with cold or flu-like symptoms (81.1%) than respondents living in Italy (90.2%) or in the Netherlands (89.0%). Regarding behaviors related to travel, respondents from Italy more often reported to have reduced the amount they went to school or work (94.4%, compared to 88.0% in the Netherlands and 84.9% in Germany, of public transport use (98.6% compared to 89.6% in the Netherlands and 91.3% in Germany), and of going to shops (97.7%, compared to 81.4% in the Netherlands and 67.5% in Germany) (Fig 3 and Table 3). Responses pertaining to limiting interactions and avoiding traveling may reflect a mixture of government-imposed restrictions and respondents’ own awareness and willingness to follow protective measures. Therefore, we additionally asked respondents whether they kept children at home prior to any formal mandates to assess the percentage of respondents that applied measures on their own accord. Of those indicating the question was applicable to their situation, in Italy, 60.6% kept their children at home before isolation measures were enacted compared to 53.6% in the Netherlands and 58.1% in Germany.

### Subgroup analyses among respondents living in the Netherlands

Although we conducted no formal comparisons between the sociodemographic subgroups of participants, some patterns were evident among respondents living in the Netherlands (S3 Appendix). In general, while there were no substantial differences in the degrees of belief in the effectiveness of protective measures between gender groups, participants identifying as women reported having applied these measures most frequently. Among the different age groups, the belief in the effectiveness of a complete social lockdown differed (e.g. ≤ 20 years: 47.5%, 21–40 years: 62.7%). Differences were also observed among subgroups with different daily activities (e.g. retired: 55.4%, homemaker/unemployed: 64.7%) and different education levels (e.g. primary/secondary: 54.6%, tertiary academic: 61.1%). Chronically ill patients more frequently reported exhibiting protective behaviors than respondents without any chronic diseases. Different sociodemographic subgroups used different sources of information to obtain information related to the COVID-19 pandemic. With higher age, the percentage of respondents who agreed they felt sufficiently informed was higher (e.g. ≤ 20 years: 87.0%, versus > 60 years: 94.9%).

### Change in responses over time

In the week immediately following the primary data collection period, we received responses from 1,588 additional participants, of whom 858 reported living in the Netherlands, 413 in Germany, and 94 in Italy. There were no changes in stages of community containment measures in the three countries throughout the additional week of data collection according to the classification system (S2 Appendix). In general, we observed no substantial changes in the aggregate responses over time (S4 Appendix), except for a decrease in the belief in the effectiveness of a complete social lockdown in Germany (S4 Appendix). Furthermore, among respondents from the Netherlands, we observed a small increase in the percentages of respondents indicating they believe in the effectiveness of preventive measures (range: 0%-5%) and those indicating they implemented these measures (range: 0%-10%) across both data collection periods (S4 Appendix).

## Discussion

Our findings indicate that in three European countries, the Netherlands, Germany, and Italy, the public belief in the effectiveness and the actual implementation of certain protective measures during the early phase of the COVID-19 pandemic in March 2020 was high. Furthermore, residents reported to be sufficiently informed about the ongoing pandemic using various communication channels.

In mid-March, the public belief in the effectiveness of protective measures was highest among respondents residing in Italy, which had the most extensive measures of social lockdown as well as the highest numbers of COVID-19 cases and deaths in Europe during the study period. Compared to the Netherlands and Germany, respondents living in Italy most often reported not only exhibiting behaviors related to government-imposed restrictions but also voluntary hygienic and social measures. Although more than 90% of respondents indicated belief in the effectiveness of imposed measures of social distancing, a complete social lockdown was deemed effective by only 59% of respondents residing in the Netherlands (compared to 77% in Germany and 87% in Italy). At the time of survey completion, comparatively looser social distancing measures were enforced there. The results of our study suggest that the level of community containment measures implemented by national governments may be rapidly visible in the public beliefs about protective measures, the extent to which people actually exhibit these relevant behaviors, and reflect the severity of the outbreak situation in a given country.

Since the initiation of this study, more research is published on this topic. Results from two recently published survey studies conducted in the USA, the UK, and China primarily focus on the respondents’ knowledge about COVID-19 and assess understanding pertaining to the disease course [17,18]. Three studies in the USA found that people had a relatively low perception of risk posed by the COVID-19 pandemic [9,19] and that vulnerable communities in society, such as those with low health literacy, living below poverty level, and racial minority groups, were often not well informed about COVID-19, perceived less risk and initiated fewer preventive behaviors [10].

We found that traditional information sources (e.g. television and news) were used most frequently among our respondents. A study conducted between January 24th and February 13th, 2020 among 1715 Hong Kong residents showed that most respondents there obtained information on the COVID-19 pandemic from social media and websites [20]. In addition, McFadden et al. showed that people primarily prefer health officials and professionals as a source of information on COVID-19 and least prefer social media, and friends and family[9].

### Social distancing and other behavioral measures

As the transmissibility of the SARS-CoV-2 virus is estimated to be similar to or higher than previous coronaviruses such as SARS, social isolation measures are particularly important [21,22]. Social (physical) distancing has been proposed as one of the most effective measures for mitigating pandemics caused by viruses, including COVID-19 [5,8,23]. In the current COVID-19 pandemic, models have shown that the Wuhan quarantine reduced transmission of COVID-19 cases from mainland China to other countries by 77% by early February [24]. Besides social distancing, other behavioral protective measures have also been deemed effective in the mitigation of the current pandemic. For instance, regular hand washing may result in a reduction of peak infection rate up to 65% with a delay of 2.7 months and a 29% decrease in total infection rate [25].

Generally, preventive, precautionary behavior is more commonly observed, among women, and in older persons [26–28], which was also reflected in our findings from Europe during the ongoing COVID-19 pandemic. In a recent survey study in the USA, greater risk perception for COVID-19 infection and infection fatality resulted in a greater likelihood of implementing protective behaviors [19]. To promote the implementation of preventive behaviors, COVID-19 risks perception may need to be addressed in communications to increase awareness.

Although our survey primarily focused on enacted governmental measures, it implicitly covered voluntary, self-initiated measurements taken by respondents. Previous pandemics have shown that people appear to respond by voluntarily engaging in preventive behaviors. During the 2003 SARS epidemic, Hong Kong and Beijing residents showed voluntary behavioral change in response to disease outbreak, such as avoiding local and international transport and public places [29,30]. During the 2009 Influenza A (H1N1) pandemic, people from the USA voluntarily reduced their time spent in public spaces [31] and many passengers opted not to travel [32]. During the COVID-19 pandemic, time spent at home increased even before restrictive governmental policies were enacted [33] and government-imposed social distancing measures decreased daily public transport use [34]. Voluntary behavioral responses of the overall population may play an important role within the control of a pandemic.

### Provision and acquirement of information during pandemics

Transparent, timely, and easy-to-understand information is essential to increase trust in national governments during pandemics [35]. The increasing use of portable devices and social media is evident in our findings, which indicate frequent use of social media to acquire pandemic-related information (range across countries: 40.0%-55.8%). However, in recent epidemics and pandemics, a substantial amount of online information, especially distributed via social media, was found to be incorrect and misleading [36,37]. Environmental cues to follow behavioral recommendations, favorable attitudes towards prevention measures, and knowledge about the virus were associated with exhibiting protective behavior [5]. Therefore, accurate information provision via social media channels is crucial, besides information via traditional information sources.

### Study strengths and limitations

Given the evolving pandemic situation, we felt it was important to develop, translate, and disseminate our questionnaire rapidly during the early phase in order to capture a snapshot of public perceptions and behaviors as the COVID-19 crisis unfolded and formal containment measures were enacted in several European countries. Many items in our survey were adapted from an existing validated questionnaire created to assess perceptions and behaviors in response to influenza [12]. We attempted to make our survey accessible to participants of diverse backgrounds by providing the survey in eight languages. These translations could be readily adapted for use in future outbreaks.

Readers should consider some important limitations when interpreting our findings. First, since the survey was web-based and recruitment was largely through digital channels including social media, we acknowledge the potential for selection bias. We cannot assume that our study population is representative for the individual countries and acknowledge possible over-representation of health-conscious individuals and those more concerned about the outbreak. In addition, as the survey was distributed widely through social media, its response rate could not be ascertained, as the exact denominator is unknown. However, under the exceptional current circumstances, members of the general public who normally would not participate in health-related surveys may have been more likely to participate given the media attention, severity, and the pandemic’s large impact on many aspects of daily life. Furthermore, with the social (physical) distancing recommendations and enforced measures in place during this period, many were confined to their homes turned to social media and other web-based platforms for social communication, including older individuals. Second, the number of completed survey responses was much higher among residents of the Netherlands compared to Italy or Germany. This is unsurprising since the majority of our research team members are based in the Netherlands and the largest dissemination efforts occurred there. Third, while the governments of Germany, Italy, and the Netherlands enacted different mitigation measures in each country, our survey was not adapted to reflect country-specific nuances. Hence, we acknowledge that our results might not fully depict whether residents of these countries actually followed their country-specific measures. Fourth, we cannot rule out that an individual completed the survey more than once on multiple devices, in another browser, or by clearing cookies; however, repeat submissions from the same device were not accepted.

Fifth, with regard to the secondary analysis over the extended data collection period, we observed no major changes in the aggregated answers over time; however, we acknowledge a possible delay between the implementation of formal community isolation measures and the subsequent information uptake and application of these measures by residents. During the extended data collection period, response rates were lower than in the primary collection period, especially from respondents living in Italy. Since the survey was administered cross-sectionally, the changes in responses observed over time could be attributed to differences in respondents' characteristics.

Finally, we emphasize the aim of our study was descriptive, and we caution readers to interpret the data accordingly. A formal comparison between countries would require appropriate analytical consideration of variables taking into account sociocultural (including educational systems), political, and structural contexts in each country. In an effort to help the reader better understand each country-specific setting we included information about the stage of containment measures enacted within the included countries in a timeline encapsulating the data collection period.

## Conclusion

The extent to which individuals internalize and respond to (government-mandated) mitigation measures and recommendations is of interest to the control of the spread of the SARS-CoV-2 virus and to optimize outcomes during the current COVID-19 pandemic. In our survey study of the general public living in the Netherlands, Germany, and Italy, we found that approval and application of publicly enforced and self-initiated protective measures during the early pandemic phase were highest in Italy, the region with the most extensive measures of social lockdown and highest burden (number of cases and deaths) in Europe in mid-March 2020,Media channels used to acquire information and the extent to which respondents felt sufficiently informed about the COVID-19 pandemic differed per country and among sociodemographic subgroups in the Netherlands. No substantial changes in the perceived effectiveness of behavioral protective measures and the implementation of these measures in these countries were observed between March 19th and March 30th, 2020 as the COVID-19 pandemic continued to evolve in Europe and formal community isolation measures became stricter. We believe these insights are valuable to inform the information dissemination and infection control strategies of governments and public health organizations during the current crisis and for future pandemics.

## Supporting information

S1 Checklist STROBE 2007 (v4) statement—checklist of items that should be included in reports of cross-sectional studies. (DOCX)

S1 Appendix Survey in all languages. (PDF)

S2 Appendix Daily stage classification of community containment measures taken by country in March 2020. (PDF)

S3 Appendix Subgroup analyses among respondents living in the Netherlands. (PDF)

S4 Appendix Perceived effectiveness of behavioral protective measures and the implementation of these measures in the Netherlands, Germany and Italy between March 19th and March 30th, 2020. (PDF)
