# Supplementary material for: Public perspectives on protective measures during the COVID-19 pandemic in the Netherlands, Germany and Italy: A survey study
Source: PLoS One. 2020 Aug 5;15(8):e0236917. doi: 10.1371/journal.pone.0236917 (PMC7406072; doi:10.1371/journal.pone.0236917)
Supplement: S1 Appendix — (PDF) [file pone.0236917.s002.pdf]

S1 Appendix 1. Survey in all languages.

English

Q1. Which of the following sources have you primarily used in the past 7 days to acquire information on the recent COVID-19 outbreak? Select a maximum of 3 sources.

- Television (e.g. news)
- Newspaper, mobile news application
- Social media (e.g. YouTube, Facebook, Twitter, Instagram, Reddit, LinkedIn)
- Radio
- Official health hotlines (e.g. NHS)
- Official health websites (e.g. WHO)
- Healthcare professionals (e.g. doctor, GP, pharmacist, other)
- People I speak to on a daily basis (e.g. family, friends, colleagues)

Q2. Please indicate to what extent you agree with the following statement.

- I have been informed sufficiently about the recent outbreak of COVID-19 and about what I can do to avoid being infected: probably true, probably false, not sure, no opinion

Q3 The following statements refer to possible protective behaviours that you may or may not have exhibited to prevent further spread of the COVID-19 outbreak. Please indicate whether you have or have not applied these measures in the past 7 days due to the viral outbreak: yes, no, not sure, or not applicable

- Reduced the amount you go to school, college, university or work
- Cancelled or postponed a social event such as meeting friends, eating out or going to a sports event
- Reduced the use of or changed the way you use public transport
- Kept one or more of your children out of school or daycare voluntarily before any mandates were put in place
- Reduced the amount you go to shops
- Kept away from crowded places
- Cleaned or disinfected things you might touch (such as door knob or hard surfaces) more often than usual
- Carried sanitizing hand gel with you when out and about
- Used sanitising hand gel to clean your hands, more often than usual
- Reduced the amount you touch your eyes, nose and/or mouth
- Followed a healthy diet or took vitamin supplements
- Tried to avoid people who have the cold or flu-like symptoms
- Usually carried tissues with you when out and about
- Usually used tissues when sneezing or coughing
- Washed your hands with soap and water more often than usual
- Have you done anything else to avoid catching flu that was not already mentioned?

Q4. The following statements concern measures to prevent further spread of COVID-19. Indicate for each of the following statements whether you think they are: probably true, probably false, or if you don't know.

An effective way to reduce outspread is:

- Avoiding social gatherings
- Selective closure of public places/locations

- Implementation of hand hygiene measures
- Implementation of respiratory measures
- Complete social lockdown/isolation

The following questions concern information about your sociodemographic background. Please select the option that is applicable to your situation.

Q5. What is your gender?
- Male
- Female
- Not listed

Q6. What is your age?
- ≤20 years
- 21-30 years
- 31-40 years
- 41-50 years
- 51-60 years
- 61-70 years
- 71-80 years
- >80 years

Q7. What is your primary daily activity?
- Paid job
- Homemaker
- Student
- Retired
- Unemployed
- Other

Q8. What is your current household composition?
- Single
- Single parent with child(ren)
- Living with partner
- Living with partner and child(ren)
- Shared flat (with roommates)
- Other

Q9. What is your highest educational qualification?
- Primary
- Secondary
- Tertiary vocational
- Tertiary academic

Q10. Are you a healthcare provider (e.g. doctor, nurse) or (bio)medical student?
- Yes
- No

Q11. In which country do you currently reside?

Q12. Do you suffer from any chronic illness or poor medical condition?
(e.g. respiratory disease, heart disease, metabolic disorders such as diabetes, (previous) cancer treatment or other diseases requiring chronic medication)

- Yes
- No

Q13. By using which social channel did you open this survey?

- Facebook
- Twitter
- Instagram
- LinkedIn
- WhatsApp
- National news/news fora
- Other:

Dutch

Q1 Welke van de volgende bronnen heeft u in de afgelopen 7 dagen het meest gebruikt om informatie over de recente uitbraak van COVID-19 te verkrijgen? Kies maximaal 3 bronnen.

- Televisie (o.a. nieuwsprogramma's)
- Kranten, nieuws applicaties
- Sociale media (o.a. YouTube, Facebook, Twitter, Instagram, Reddit, LinkedIn)
- Radio
- Telefonisch via officiële gezondheidsinstanties (bijv. RIVM, GGD, Rode Kruis)
- Websites van officiële gezondheidsinstanties (bijv. RIVM, GGD, Rode Kruis)
- Zorgverleners (bijv. (huis)arts, apotheker, doktersassistent)
- Familie, vrienden, collega's met wie ik dagelijks praat

Q2. Geef aan in hoeverre u het eens bent met de volgende stelling.
Ik ben voldoende geïnformeerd over de recente uitbraak van COVID-19 en over wat ik kan doen om verdere verspreiding te voorkomen: waarschijnlijk waar, waarschijnlijk niet waar, onzeker, geen mening

Q3. **De volgende uitspraken gaan over bepaalde handelingen die u mogelijk heeft ondernomen ter preventie van verdere verspreiding van COVID-19. Geef aan of u deze wel of niet heeft toegepast in de afgelopen 7 dagen vanwege de virusuitbraak: ja, nee, onzeker, niet van toepassing**

- Minder vaak naar (hoge) school, universiteit of werk gaan
- Afzeggen of uitstellen van sociale bijeenkomsten zoals afspreken met vrienden, uit eten gaan of een sportevenement bezoeken
- Minder vaak, of op een andere manier, gebruik maken van openbaar vervoer
- Eén of meer van uw kinderen niet naar school of dagverblijf gebracht voordat dit verplicht werd
- Minder vaak winkels bezoeken
- Vermijden van drukke plekken

- Voorwerpen die u aanraakt vaker dan gebruikelijk schoonmaken of desinfecteren (zoals deurknoppen of gladde oppervlakken)
- Ontsmettende handgel bij u dragen als u op pad bent
- Vaker dan gebruikelijk uw handen schoon maken met ontsmettende handgel
- Minder vaak uw ogen, neus en/of mond aanraken
- Een gezond dieet volgen of vitaminesupplementen innemen
- Proberen personen met verkoudheids- of griepklachten te vermijden
- Papieren zakdoekjes bij u dragen als u op pad bent
- Regelmatig gebruik maken van papieren zakdoekjes bij niezen of hoesten
- Vaker dan gebruikelijk uw handen gewassen met water en zeep
- Heeft u andere maatregelen die nog niet genoemd zijn genomen, om besmetting met het virus te vermijden?

Q4. De volgende stellingen gaan over maatregelen ter preventie van verdere verspreiding van COVID-19. Geef voor elk van de volgende stellingen aan of u denkt dat deze: waarschijnlijk waar zijn, waarschijnlijk onwaar zijn of dat u het niet zeker weet.

Een effectieve manier om verspreiding te voorkomen is:
- Vermijden van sociale bijeenkomsten
- Het selectief sluiten van openbare plekken en locaties
- Invoeren van handhygiëne maatregelen
- Invoeren van maatregelen ter voorkoming van besmetting via de luchtwegen (bijv. praten vanaf 2 meter afstand, in elleboog/zakdoek hoesten/niezen)
- Volledige sociale isolatie

De volgende vragen hebben betrekking op u. Vink de optie aan die voor u van toepassing is.

Q6. Wat is uw geslacht?
- Man
- Vrouw
- Anders:

Q7. Wat is uw leeftijd?
- ≤20 jaar
- 21-30 jaar
- 31-40 jaar
- 41-50 jaar
- 51-60 jaar
- 61-70 jaar
- 71-80 jaar
- >80 jaar

Q8. Wat is uw voornaamste dagelijkse bezigheid?
- Betaald werk
- Huisvrouw/huisman
- Student
- Gepensioneerd
- Werkloos

- Anders

Q9. Wat is de samenstelling van uw huishouden?
- Alleenstaand
- Alleenstaand met kind(eren)
- Samenlevend met partner
- Samenlevend met partner en kind(eren)
- Samenlevend met huisgenoten
- Anders

Q10. Wat is uw hoogst genoten opleiding?
- Basisonderwijs
- MAVO/ VMBO (België: BSO)
- MBO/ HAVO/ VWO (België: ASO, TSO, KSO)
- HBO/ WO (Hogeschool, Universiteit)

Q11. Bent u een zorgverlener (bijv. arts, verpleegkundige) of (bio)medische student?
- Ja
- Nee

Q12. In welk land woont u op dit moment?

Q13. Heeft u een chronische ziekte of een verminderde gezondheidstoestand?
(bijv. chronische longziekten, chronische hartziekten, metabole ziekten zoals diabetes, onder behandeling (geweest) voor kanker of iets anders waarvoor chronische medicatie nodig is)
- Ja
- Nee

Q14. Via welk medium bent u bij deze vragenlijst terecht gekomen?
- Facebook
- Twitter
- Instagram
- LinkedIn
- WhatsApp
- Nationaal nieuws / nieuwsfora
- Anders:

French

Q1. Parmi les sources suivantes, lesquelles avez-vous consultées le plus fréquemment au cours des 7 derniers jours pour obtenir des informations sur la récente épidémie de COVID-19? Veuillez cocher un maximum de 3 sources.
- Télévision (par exemple, programmes d'information)
- Journaux, applications d'information
- Médias sociaux (YouTube, Facebook, Twitter, Instagram, Reddit, LinkedIn)
- Radio

- Autorités sanitaires officielles par téléphone
- Sites web officiels des autorités sanitaires
- Les personnes qui s'occupent de l'enfant (par exemple, le médecin (de famille), le pharmacien)
- Contacts sociaux personnels (par exemple au travail, à l'école)

Q2. Indiquez dans quelle mesure vous êtes d'accord avec la déclaration suivante.
Je suis suffisamment informé sur la récente épidémie de COVID-19 et sur ce que je peux faire pour empêcher sa propagation: probablement vrai, probablement pas vrai, incertain, pas d'avis

Q3. Les déclarations suivantes concernent certaines actions que vous avez pu entreprendre pour empêcher la diffusion de COVID-19. Veuillez indiquer si vous les avez appliquées ou non au cours des 7 derniers jours en raison de l'apparition du virus: oui, non, incertain, pas d'application
- Aller moins souvent à l'école, à l'université ou au travail
- Annuler ou reporter des rencontres sociales telles que des rendez-vous avec des amis, un dîner ou une visite dans un club (de sport)
- Utiliser les transports publics moins fréquemment ou d'une autre manière
- Un ou plusieurs de vos enfants n'ont pas été amenés à l'école ou à la crèche avant que cela ne devienne obligatoire
- Visiter moins souvent les magasins
- Éviter les zones de surpopulation en général
- Nettoyer ou désinfecter les objets que vous touchez plus souvent que d'habitude (comme les poignées de porte ou les surfaces dures)
- Apporter/transporter des produits d'hygiène des mains plus souvent que d'habitude
- Utiliser les produits d'hygiène des mains plus fréquemment que d'habitude
- Se toucher les yeux, le nez et/ou la bouche moins souvent
- Suivre un régime alimentaire sain ou prendre des suppléments vitaminiques
- Éviter les personnes présentant des symptômes de rhume ou de grippe
- Porter des mouchoirs plus souvent que d'habitude
- Utilisation régulière de mouchoirs en papier pour éternuer ou tousser
- Se laver les mains à l'eau et au savon plus souvent que d'habitude
- Autres mesures de prévention de l'infection par le virus non mentionnées

Q4. Les déclarations suivantes concernent les mesures visant à prévenir la propagation de la COVID-19. Pour chacune des déclarations suivantes, indiquez si vous pensez qu'elles sont: probablement vraies, probablement fausses ou incertaines.

Un moyen efficace de prévenir la prolifération est de...

- Éviter les rassemblements sociaux (par exemple les célébrations)
- Fermeture sélective de lieux publics (tels que les établissements de restauration)
- Application des mesures d'hygiène des mains
- Appliquer des mesures visant à prévenir la contamination par des particules virales libérées (par exemple, parler à une distance de 2 mètres, tousser / éternuer dans le coude / le mouchoir)
- Isolement social complet

Les questions suivantes se rapportent à votre situation actuelle. Veuillez cocher l'option de réponse qui vous convient le mieux.

Q5. Quel est votre sexe?
- Homme
- Femme
- Sinon:

Q6. Quel est votre âge?
- ≤20 ans
- 21-30 ans
- 31-40 ans
- 41-50 ans
- 51-60 ans
- 61-70 ans
- 71-80 ans
- >80 ans

Q7. Quelle est votre principale activité quotidienne?
- Travail rémunéré
- Femme/homme au foyer
- Étudiant
- Retraités
- Chômeurs
- Sinon

Q8. Quelle est la composition de votre ménage?
- Unique
- Parent seul avec enfants
- Couple
- Couple avec enfant(s)
- Appartement partagé (avec colocataire(s))
- Sinon

Q9. Quel est votre niveau d'études le plus élevé?
- Primaire
- Baccalauréat
- Etudes techniques et professionnelles
- Etudes académiques

Q10. Êtes-vous un professionnel de la santé (p. ex. médecin, infirmière) ou étudiant en (bio)médecine?
- Oui
- Non

Q11. Dans quel pays vivez-vous actuellement?

Q12. Vous souffrez d'une maladie chronique ou vous êtes dans un état de santé réduit?
(par exemple, les maladies pulmonaires chroniques, les maladies cardiaques chroniques, les maladies métaboliques telles que le diabète, le traitement du cancer ou toute autre maladie nécessitant une médication chronique)

- Oui
- Non

Q13. Par quel moyen êtes-vous entré en contact avec ce questionnaire?

- Facebook
- Twitter
- Instagram
- LinkedIn
- WhatsApp
- Forums d'information nationaux
- Sinon:

German

Q1. Welche der folgenden Informationsquellen haben Sie in den letzten 7 Tagen am meisten genutzt um Informationen über den COVID-19 Ausbruch zu erhalten? Wählen Sie maximal 3 Antworten aus.

- Fernsehen (z.B. Nachrichten)
- Zeitung, Zeitungsapps
- Soziale Medien (z.B. Youtube, Facebook, Twitter, Instagram, Reddit, LinkedIn)
- Radio
- Offizielle Telefonhotline (z.B. von Behörden oder Krankenkassen)
- Offizielle Webseiten (z.B. des Robert-Koch-Instituts, der Weltgesundheitsorganisation)
- Gesundheitsdienstleister (z.B. Ärzt*innen, Hausärzt*innen, Pharmazeut*innen, Apotheker*innen, andere)
- Menschen mit denen ich eh im Kontakt stehe (z.B. Familie, Freund*innen, Kolleg*innen)

Q2. Bitte geben Sie an inwiefern Sie der folgenden Aussage zustimmen: vermutlich, vermutlich nicht, nicht sicher, keine Meinung

- Ich wurde ausreichend über den COVID-19 Ausbruch informiert und auch wie ich mich vor einer Infektion schützen kann.

Q3. Die folgenden Aussagen betreffen potenziell schützende Verhaltensweisen, die Sie evtl. umsetzen oder nicht. Bitte geben Sie an, ob Sie die folgenden Verhaltensweisen in den letzten 7 Tagen wegen des COVID-19 Ausbruches angewendet haben: ja, nein, nicht sicher, nicht zutreffend

- Weniger in die Schule, Universität oder zur Arbeit gegangen
- Soziale Ereignisse wie z.B. Freund*innen treffen, Essen gehen oder Besuch von Sportveranstaltung abgesagt oder verschoben
- Die Nutzung des öffentlichen Nahverkehrs reduziert oder verändert
- Eines oder mehrere Kinder freiwillig zu Hause behalten bevor es zu Kita- bzw. Schulschließungen kam

- Weniger einkaufen gegangen
- Von belebten Plätzen ferngehalten
- Gegenstände, die man gewöhnlich anfasst öfter als normal desinfiziert oder gesäubert (z.B. Türklinken oder Oberflächen)
- Handdesinfektionsmittel für unterwegs mitgenommen
- Hände öfter als gewöhnlich desinfizieren
- Weniger in die Augen, Nasen und/oder Mund gefasst
- Sich gesund ernährt oder Nahrungsergänzungsmittel genommen
- Kontakt zu Menschen mit Erkältungssymptomen vermieden
- Taschentücher für unterwegs mitgenommen
- Taschentücher benutzt, wenn man geniest oder gehustet hat
- Hände öfter als gewöhnlich mit Wasser und Seife gewaschen.
- Haben Sie andere Maßnahmen angewendet die noch nicht genannt worden sind?

Q4 Die folgenden Aussagen behandeln Verhaltensweisen, die eine Weiterverbreitung von COVID-19 verhindern sollen. Geben Sie für jede Aussage an, ob Sie diese als vermutlich wahr, vermutlich falsch oder nicht wahr erachten, oder ob Sie dazu keine Meinung haben.

Eine effiziente Methode die Weiterverbreitung zu vermeiden ist:

- Vermeidung von sozialen Zusammenkünften
- Selektive Schließung von öffentlichen Plätzen/Lokalitäten
- Anwendung von Maßnahmen zur verbesserten Handehygiene
- Anwendung von Maßnahmen bezüglich des Atmungsapparates (z.B. größere Distanz beim Reden, Niesen/Husten in die Armbeuge/Taschentuch)
- Ausgangssperren und komplette Isolation

Die folgenden Fragen beziehen sich auf Ihre sozialen und demographischen Lebensverhältnisse. Bitte wählen Sie die Antwort, die Ihre Situation am besten beschreibt.

Q5. Was ist Ihr Geschlecht?

- Männlich
- Weiblich
- Andere:

Q6. Wie alt sind Sie?

- ≤20 Jahre
- 21-30 Jahre
- 31-40 Jahre
- 41-50 Jahre
- 51-60 Jahre
- 61-70 Jahre
- 71-80 Jahre
- >80 Jahre

Q7. Was ist Ihre Haupttätigkeit?

- Berufstätig
- Im Haushalt tätig
- Student*in

- Rentner*in
- Arbeitslos
- Andere

Q8. In was für einem Haushalt leben Sie?
- Single
- Alleinerziehend mit Kind/ern
- In einer Partnerschaft
- In einer Partnerschaft mit Kind/ern
- In einer Wohngemeinschaft
- Andere

Q9. Was ist Ihr höchster Bildungsabschluss?
- Haupt-/Realschule
- Abitur
- Berufsschule
- Hochschule/Universität

Q10. Sind sie ein Mitglied des Gesundheitssystems (z.B. Mediziner*in, Krankenpfleger*in) oder ein*e Student*in der (Bio-)Medizin?
- Ja
- Nein

Q11. In welchem Land leben Sie?

Q12. Leiden oder litten Sie an chronischen Erkrankungen oder schlechter Gesundheit?
(*z.B. Atemwegserkrankungen, Herzkrankheiten, Stoffwechselkrankheiten wie Diabetes, Krebs, oder andere Krankheiten, die medikamentös behandelt werden*)
- Ja
- Nein

Q13. Wie sind Sie auf diese Umfrage aufmerksam geworden?
- Facebook
- Twitter
- Instagram
- LinkedIn
- WhatsApp
- Nachrichten
- Andere:

Italian

Q1. Quali dei seguenti canali hai utilizzato negli ultimi 7 giorni per informati circa la diffusione del COVID-19? Seleziona massimo 3 opzioni.

- Televisione (es. telegiornali)
- Quotidiani cartacei o online
- Social media (YouTube, Facebook, Twitter, Instagram, Reddit, LinkedIn)
- Radio
- Numeri verdi nazionali o regionali (es. 1500)
- Siti web ufficiali (es. sito dell'Organizzazione Mondiale della Sanità, sito del Ministero della Salute)
- Personale sanitario (es. medico di famiglia, medico specialista, farmacista, altro)
- Persone con cui parli quotidianamente (es. familiari, amici, colleghi)

Q2. Indica in quale misura sei d'accordo con la seguente affermazione: abbastanza d'accordo, abbastanza in disaccordo, non so, nessuna opinione

- Sono stato sufficientemente informato sulla recente epidemia di COVID-19 e su cosa posso fare per evitare di essere contagiato

Q3. Le seguenti affermazioni riguardano comportamenti adottabili per prevenire un'ulteriore diffusione del COVID-19. Indica se hai applicato o meno queste misure negli ultimi 7 giorni a causa dell'epidemia virale: si, no, non so, non pertinente.

- Ridurre il tempo trascorso a scuola/ università/ lavoro
- Cancellare o rimandare eventi sociali come incontrare amici, mangiare fuori o partecipare a eventi sportivi
- Modificare o ridurre l'utilizzo dei mezzi di trasporto pubblico
- Decidere di non mandare uno o più bambini a scuola o all'asilo anche prima che venissero implementate misure restrittive
- Ridurre la frequenza con cui ti rechi nei negozi
- Evitare luoghi affollati
- Pulire e disinfettare più frequentemente gli oggetti con cui vieni in contatto (maniglie delle porte, superfici rigide)
- Portare con te un gel igienizzante per le mani quando esci di casa
- Utilizzare il gel igienizzante per le mani più spesso del solito
- Ridurre la frequenza con cui ti tocchi gli occhi, il naso e la bocca
- Seguire una dieta più sana o assumere integratori di vitamine
- Provare a evitare contatti con persone raffreddate o che presentano sintomi influenzali
- Portare con te fazzoletti di carta quando esci di casa
- Usare fazzoletti di carta quando starnutisci o tossisci
- Lavare le mani con il sapone più frequentemente del solito
- Per evitare di ammalarti, hai adottato altre precauzioni non menzionate nella lista?

Q4. Le affermazioni seguenti riguardano alcune misure per prevenire la diffusione del COVID-19. Per ognuna delle seguenti affermazioni, indica se sei: abbastanza d'accordo, abbastanza in disaccordo o se non sai.

Un modo efficace per ridurre il contagio è...

- Evitare assembramenti
- Chiudere alcuni locali e luoghi pubblici
- Prestare maggiore attenzione all'igiene delle mani
- Prestare maggiore attenzione alle buone pratiche igieniche nei rapporti ravvicinati (es. parlare a debita distanza, starnutire/tossire nel gomito o in un fazzoletto)
- Quarantena totale (isolamento)

Le seguenti domande riguardano informazioni sulla tua situazione socio-demografica. Scegli l'opzione più adatta a te.

Q5. Qual è il tuo genere?
- Uomo
- Donna
- Altro:

Q6. Qual è la tua età?
- ≤20 anni
- 21-30 anni
- 31-40 anni
- 41-50 anni
- 51-60 anni
- 61-70 anni
- 71-80 anni
- >80 anni

Q7. Qual è la tua principale attività?
- Lavoratore(-trice)
- Casalingo(a)
- Studente(-ssa)
- Pensionato(a)
- Disoccupato(a)
- Altro

Q8. Com'è composto attualmente il tuo nucleo familiare?
- Single
- Genitore single con bambino/i
- Convivenza con partner
- Convivenza con partner e bambino/i
- Appartamento condiviso (con coinquilini)
- Altro

Q9. Qual è il tuo titolo di studio?
- Licenza elementare
- Licenza media
- Diploma di maturità
- Laurea

Q10. Sei un operatore sanitario (es. medico, infermiere) o uno studente di medicina?
- Si
- No

Q11. In che Paese vivi?

Q12. **Soffri di malattie croniche e/o hai particolari condizioni di salute?**
(es. malattie respiratorie, malattie cardiache, disturbi metabolici come il diabete, trattamenti oncologici in corso o svolti precedentemente, altre malattie per le quali è necessario l'uso costante di farmaci)
- Si
- No

Q13. Tramite quale canale sei venuto a conoscenza del presente questionario?
- Facebook
- Twitter
- Instagram
- LinkedIn
- WhatsApp
- Giornali nazionali/forum di attualità
- Altro:

Spanish

Q1. ¿Cuál de las siguientes fuentes ha utilizado principalmente en los últimos 7 días para obtener información sobre el reciente brote de COVID-19? Seleccione un máximo de 3 fuentes.
- Televisión (por ejemplo, noticias)
- Periódico, aplicación de noticias móvil
- Redes sociales (por ejemplo, YouTube, Facebook, Twitter, Instagram, Reddit, LinkedIn)
- Radio
- Líneas directas de salud oficiales (por ejemplo, Ministerio de Sanidad, Consumo y Bienestar Social, Consejería de Salud de Comunidades Autónomas)
- Sitios web oficiales de salud (por ejemplo, OMS)
- Profesionales de la salud (por ejemplo, médico de cabecera, farmacéutico u otro)
- Personas con las que hablo a diario (por ejemplo, familiares, amigos, colegas)

Q2. Indique en qué medida está de acuerdo con la siguiente declaración: probablemente cierto, probablemente falso, no estoy Seguro, no tengo una opinión
- Me han informado lo suficiente sobre el reciente brote de COVID-19 y sobre lo que puedo hacer para evitar ser infectado

Q3. Las siguientes declaraciones se refieren a posibles comportamientos de protección que puede haber exhibido o no para evitar una mayor propagación del brote de COVID-19. Indique si ha aplicado o no estas medidas en los últimos 7 días debido al brote viral: si, no, no estoy seguro, no procede
- Redujo la cantidad de tiempo que vas a la escuela, colegio, universidad o trabajo
- Canceló o pospuso un evento social como reunirse con amigos, salir a comer o ir a un evento deportivo
- Redujo el uso o cambió la forma de uso de transporte público
- Mantuvo a uno o más de sus hijos fuera de la escuela o guardería voluntariamente antes de que se estableciera cualquier mandato
- Redujo la cantidad de veces o tiempo que pasó en tiendas
- Se alejó de lugares abarrotados

- Limpió o desinfectó las cosas que podría tocar (como el pomo de la puerta o las superficies duras) con más frecuencia de lo habitual
- Llevó gel desinfectante para manos cuando salió fuera de casa
- Usó gel desinfectante para manos para limpiarse las manos con más frecuencia de lo habitual
- Redujo la cantidad que toca sus ojos, nariz y / o boca
- Siguió una dieta saludable o tomó suplementos vitamínicos
- Intentó evitar a personas con síntomas de resfriado o gripe
- Por lo general, llevó pañuelos de papel con usted cuando estuvo fuera de casa
- Normalmente utilizó pañuelos de papel al estornudar o toser
- Se lavó las manos con agua y jabón con más frecuencia de lo habitual
- Ha hecho algo más para evitar contraer el virus aún no mencionado

Q4. Las siguientes declaraciones se refieren a medidas para evitar una mayor propagación de COVID-19. Indique para cada una de las siguientes afirmaciones si cree que lo son: probablemente verdadero, probablemente falso o si no lo sabe.

Una forma efectiva de reducir la propagación es:
- Evitar reuniones sociales
- Cierre selectivo de lugares públicas
- Implementación de medidas de higiene de manos
- Implementación de medidas respiratorias (por ejemplo, distancia de conversación limitada, estornudos / tos en el codo / pañuelos)
- Cierre social completo / aislamiento

Las siguientes preguntas se refieren a información sobre sus antecedents sociodemográficos. Seleccione la opción que sea aplicable a su situación.

Q5. ¿Cuál es su sexo?
- Hombre
- Mujer
- Otro:

Q6. ¿Qué edad tiene?
- ≤20 años
- 21-30 años
- 31-40 años
- 41-50 años
- 51-60 años
- 61-70 años
- 71-80 años
- >80 años

Q7. ¿Cuál es tu actividad diaria principal?
- Trabajo pagado
- Tareas del hogar
- Estudiante
- Jubilado
- Desempleado

- Otro

Q8. ¿Cuál es la composición actual de tu hogar?
- Soltero
- Padre soltero con hijo (s)
- Viviendo con pareja
- Viviendo con pareja e hijo (s)
- Piso compartido (con compañeros)
- Otro

Q9. ¿Cuál es su cualificación educativa más alta?
- Educación primaria
- Educación secundaria
- Formación profesional
- Formación universitaria

Q10. ¿Es usted un proveedor de atención médica (por ejemplo, médico, enfermera) o estudiante de medicina o biomedicina?
- Sí
- No

Q11. ¿En qué país reside actualmente?

Q12. ¿Sufre de alguna enfermedad crónica o condición médica?
(p. ej., enfermedades respiratorias, enfermedades cardíacas, trastornos metabólicos como diabetes, tratamiento contra cáncer en el pasado u otras enfermedades que requieren medicación crónica)
- Sí
- No

Q13. ¿Por medio de qué canal social abrió esta encuesta?
- Facebook
- Twitter
- Instagram
- LinkedIn
- WhatsApp
- Noticias nacionales / foros de noticias
- Otro:

Polish

Q1. Z których z wymienionych poniżej źródeł korzystałeś(aś) najczęściej w ciągu ostatnich 7 dni, aby uzyskać informacje na temat wybuchu pandemii COVID-19? Wybierz maksymalnie 3 źródła.
- Telewizja (np. wiadomości)
- Dziennik informacyjny, gazeta, publikacje online
- Media społecznościowe (YouTube, Facebook, Twitter, Instagram, Reddit, LinkedIn)
- Radio
- Oficjalne infolinie służb opieki medycznej (np. NHS)
- Oficjalne strony internetowe poświęcone zdrowiu (np. WHO)
- Pracownicy służby zdrowia (np. lekarz, lekarz rodzinny, farmaceuta, aptekarz, inny)
- Ludzie, z którymi rozmawiam na co dzień (np. rodzina, przyjaciele, znajomi)

Q2. Wskaż, w jakim stopniu zgadzasz się z poniższym stwierdzeniem: raczej tak, raczej nie, nie jestem pewny(a), nie mam zdania
Zostałem(am) wystarczająco poinformowany(a) o niedawnym wybuchu pandemii koronawirusa COVID-19 i o tym, co mogę zrobić, aby uniknąć zarażenia

Q3. Poniższe stwierdzenia dotyczą możliwych działań, które możesz podjąć lub nie, aby zapobiec dalszemu rozprzestrzenianiu się pandemii COVID-19. Wskaż, czy
stosowałeś(aś) te środki w ciągu ostatnich 7 dni: tak, nie, nie mam pewności, nie dotyczy mnie
- Unikanie przychodzenia do szkoły, na studia, na uniwersytet lub do pracy
- Odwoływanie lub przekładanie spotkań towarzyskich, takich jak spotkanie ze znajomymi, jedzenie poza domem lub wyjście na wydarzenie sportowe
- Unikanie lub zmiana sposobu korzystania z transportu publicznego
- Unikanie wysłania dziecka do szkoły lub w przedszkola, przed wprowadzeniem jakichkolwiek oficjalnych zarządzeń
- Zmniejszenie częstotliwości zakupów w sklepach stacjonarnych
- Unikanie zatłoczonych miejsc

- Czyszczenie lub dezynfekcja powierzchni, które można dotknąć (takich jak klamki lub blaty) częściej niż zwykle
- Noszenie ze sobą żelu antybakteryjnego poza domem
- Używanie żelu antybakteryjnego do odkażania rąk częściej niż zwykl
- Unikanie dotykania oczu, nosa i/ lub ust
- Stosowanie zdrowej diety lub przyjmowanie suplementów witaminowych
- Unikanie towarzystwa osób, które mają objawy przeziębienia lub grypy
- Noszenie ze sobą chusteczek higienicznych, gdy jest się poza domem
- Używanie chusteczek higienicznych w przypadku kichania lub kaszlu
- Częstsze mycie rąk mydłem i wodą
- Czy zrobiłeś(aś) coś jeszcze, aby uniknąć zachorowania, o czym nie wspomniano powyżej?

Q4. Poniższe stwierdzenia dotyczą środków zapobiegających rozprzestrzenianiu się COVID-19. Wskaż dla każdego z poniższych stwierdzeń, czy uważasz, że są:
raczej prawdziwe, raczej fałszywe lub nie wiesz.

Skutecznym sposobem ograniczenia rozprzestrzeniania się wirusa jest:
- Unikanie spotkań towarzyskich
- Selektywne zamykanie miejsc publicznych
- Stosowanie środków higieny rąk
- Przestrzeganie zasad związanych z ograniczeniem rozprzestrzeniania się wirusa drogą powietrzną (np. zwiększenie odległości podczas rozmowy, kichanie i kasłanie w łokieć lub chusteczki higieniczne)
- Poddanie społeczeństwa całkowitej kwarantannie

Poniższe pytania dotyczą informacji o Twojej sytuacji socjodemograficznej. Wybierz odpowiedź, która najlepiej ją opisuje.

Q5. Jakiej jesteś płci?
- Mężczyzna
- Kobieta
- Nie wymieniona:

Q6. W jakim wieku jesteś?
- ≤ 20 lat
- 21-30 lat
- 31–40 lat
- 41-50 lat
- 51–60 lat
- 61–70 lat
- 71–80 lat
- > 80 lat

Q7. Czym zajmujesz się na co dzień?
- Praca
- Prowadzenie gospodarstwa domowego
- Studia
- Emerytura

- Bezrobotny(a)
- Inne

Q8. Forma gospodarstwa domowego?
- Samotny(a)
- Zamieszkuję z partnerem(ką)
- Samotny rodzic
- Para z dziećmi
- Współdzielone mieszkanie (ze współlokatorami)
- Inna:

Q9. Jakie jest Twoje wykształcenie?
- Podstawowe
- Średnie
- Zawodowe
- Wyższe

Q10. Czy pracujesz obecnie w służbie zdrowia lub studiujesz na kierunkach (bio)medycznych?
- Tak
- Nie

Q11. W jakim kraju obecnie mieszkasz?

Q12. Czy cierpisz na przewlekłą chorobę lub zły stan zdrowia?
(np. choroby układu oddechowego, choroby serca, zaburzenia metaboliczne, takie jak cukrzyca, lub inne choroby wymagające przewlekłego leczenia)
- Tak
- Nie

Q13. W jaki uzyskałeś(aś) dostęp do tego kwestionariusza?
- Facebook
- Twitter
- Instagram
- LinkedIn
- WhatsApp
- Dziennik informacyjny
- Inny

Turkish

Q1. COVID-19 ile ilgili bilgi edinmek için son 7 günde hangi haber kaynağını kullandınız? En fazla 3 kaynak seçiniz.
- Televizyon (mesela haber programları)
- Gazete, haber uygulamaları

- Sosyal media (mesela YouTube, Facebook, Twitter, Instagram, Reddit, LinkedIn)
- Radyo
- Resmi kurumların telefon hattı (mesela RIVM, GGD, Rode Kruis)
- Resmi kurumların internet sayfaları (mesela RIVM, GGD, Rode Kruis)
- Sağlık hizmeti (mesela aile doktoru, eczacı)
- Ailem, arkadaş çevresi, günlük görüştüğüm iş arkadaşlarım
- Aşağıdaki ifade ile ne kadar hemfikirsiniz.

Q2. COVID-19 pandemisi ile ilgili yeterince bilgilendirildim ve daha fazla yayılmaması için ne yapmam gerektiğini biliyorum: Doğru, Doğru değil, Emin değilim, Fikirsizim

Q3. Aşağıdaki ifadeler COVID-19'a karşı almış olabileceğiniz tedbirler ile ilgilidir. Son 7 günde hangi şekilde tedbir aldığınızı bildiriniz: Evet, Hayır, Emin değilim, Uygulanamaz

- Daha az (yüksek) okul, üniversite veya iş yerine gitmek
- Sosyal aktiviteleri ertelemek veya iptal etmek (mesela yemeğe çıkmak, arkadaşlar ile buluşmak veya spor aktivitesine katılmak gibi)
- Daha az toplu taşımaya binmek
- Çocuğunuzu veya çocuklarınızı okula veya kreş'e göndermemek
- Daha az mağazalara gitmek
- Kalabalık yerlerden uzak durmak
- Temas ettiğin yerleri daha sık temizlemek (mesela kapı kolları)
- Dezenfekte etmek için yanınızda el jeli bulundurmak
- Eskiye bakarak daha sık ellerinizi el jeli ile dezenfekte etmek
- Gözlerinizi, burnunuzu ve ağızınızı daha az ellemek
- Sağlıklı bir diyete uymak veya vitamin hapları yutmak
- Grip veya soğuk algınlığı semptomları olan kişilerle temastan kaçınmak
- Dışarı çıktığınızda yanınızda kağıt mendil bulundurmak
- Öksürürken veya hapşırırken kağıt mendil kullanmak
- Eskiye bakarak daha sık ellerinizi su ve sabun ile yıkamak
- Virüsten kaçınmak için başka bir tedbirde bulundunuz mu?

Q4. Aşağıdaki ifadeler COVID-19'un yayılmaması için alınmış olan tedbirler. Lütfen bunların ne kadar yararlı olduğunu düşündüğünüzü belirtiniz. Seçenekleriniz: doğru, doğru değil veya bilmiyorum.
Virüsün yayılmasını önlemek için alınması gereken tedbirler:

- Sosyal aktivitelere gitmemek
- Halk'a açık yerleri ve mekanları kapatmak
- El hijyeni ile ilgili tedbirler almak
- Hava vasıtasıyla bulaşmayı engellemek için tedbirler almak (mesela 1,5 metre aralıkla konuşmak, direseğe/kağıt mendile öksürmek/hapşırmak)
- Sosyal karantinaya girmek

Aşağıdaki sorular sizin ile ilgili.

Q6. Cinsiyetiniz nedir?

- Erkek
- Kadın
- Başka: ___

Q7. Yaşınız hangi yaş grubunun içinde yer alıyor?

- ≤20 yaş
- 21-30 yaş
- 31-40 yaş
- 41-50 yaş
- 51-60 yaş
- 61-70 yaş
- 71-80 yaş
- >80 yaş

Q8. Günlük hayatınızda ne ile meşgulsünüz?

- İşçi
- Ev hanımı/erkeği
- Öğrenci
- Emekli
- İşsiz
- Başka: ___

Q9. Aile durumunuz nedir?

- Yalnız yaşıyorum
- Yalnız çocuklarım ile yaşıyorum
- Eşim ile yaşıyorum
- Eşim ve çocuklarım ile yaşıyorum
- Ev arkadaşlarım ile yaşıyorum
- Başka:

Q10. Aldığınız en yüksek eğitim seviyesi nedir?

- İlk okul
- Meslek lisesi
- Anadolu lisesi
- Üniversite

Q11. Bir sağlık uzmanı (doktor, hemşire) veya tıp öğrencisi misiniz?

- Evet
- Hayır

Q12. Şu an hangi ülke'de yaşıyorsunuz?

Q13. Kronik bir hastalığınız veya düşük bir direnciniz var mı?
(mesela akciğer hastalıkları, kalp hastalıkları, şeker hastalığı, kanser gibi)

- Evet
- Hayır

Q14. Bu araştırmaya nasıl ulaştınız

- Facebook
- Twitter
- Instagram
- Linkedİn
- WhatsApp
- Haber
- Başka: ____
-

Persian

Q1.

استفاده کرده **(COVID 19)** در طول هفت روز گذشته، کدام منابع از گزینه های زیر را عمدتا برای به دست آوردن اطلاعات در مورد بحران کرونا اید؟ حد اکثر ۳ مورد انتخاب کنید.

- تلویزیون(مثلا برنامه های خبری)-
- روزنامه یا اپلیکیشن های خبری در موبایل-
- رسانه های اجتماعی (اینستاگرام، توییتر، تلگرام، لینکد این و غیره)-
- رادیو-
- سامانه های تلفنی رسمی (مثلا سامانه ی تلفنی وزارت بهداشت)-
- سامانه های اینترنتی رسمی (مثلا سامانه های وزارت بهداشت، سازمان جهانی بهداشت و غیره)-
- متخصصین بهداشت و سلامت-
- دوستان، آشنایان، همکاران-

Q2.

چقدر با جمله ی ذیل موافقید؟

من به میزان کافی درباره ی شیوع بیماری اخیر کرونا و کارهایی که میتوانم برای جلوگیری از ابتلا با این عفونت انجام دهد، آگاه شده ام.

- نسبتا موافقم

- نسبتا مخالفم
- مطمین نیستم
- نظری ندارم

Q3.

؟سوالات زیر درباره ی رفتارهای ایمنی برای جلوگیری از شیوع بیشتر ویروس کرونا است. آیا در ۷ روز اخیر این اقدامات را انجام داده اید

بله-
خیر-
مطمین نیستم-
موضوعیت ندارد-

- کاهش تردد به مدرسه، دانشگاه یا محل کار
- لغو یا به تعویق انداختن رویدهادی اجتماعی مانند ملاقات دوستان، رفتن به رستوران، حضور در رویدادهای ورزشی
- تغییر یا کاهش استفاده از وسایل نقلیه عمومی
- جلوگیری از فرستادن فرزندتان به مدرسه یا مهد کودک ،قبل از تعطیلی اجباری مدارس
- کاهش تردد به مغازه ها
- اجتناب از رفتن به اماکن شلوغ
- (بیش از حد معمول تمیز یا ضد عفونی کردن اشیایی که ممکن است لمس کنید (مانند دستگیره ی در یا سطوح آشپزخانه و غیره
- همراه داشتن ژل ضدعفونی کننده ی دست، بیرون از خانه
- استفاده ی بیش از حد معمول از ژل ضدعفونی کننده ی دست
- کاهش لمس چشم، دهان و بینی
- استفاده از رژیم غذایی سالم یا مصرف مکمل های ویتامین
- دوری از افراد دارای علایم سرماخوردگی یا آنفلوانزا
- معمولا بیرون از منزل دستمال کاغذی به همراه داشته اید
- استفاده از دستمال کاغذی حین عطسه یا سرفه
- شستن بیش از حد معمول دستها با آب و صابون
- ؟بجز موارد بالا، آیا کار دیگری برای جلوگیری از ابتلا به آنفلوانزا انجام داده اید

Q4.

جملات زیر راجع به اقدامات پیشگیرانه برای جلوگیری از شیوع بیشتر ویروس کرونا است. لطفا نظر خود را راجع به هر کدام از اقدامات با انتخاب .یکی از سه گزینه اعلام بفرمایید

احتمالا درست-
احتمالا نادرست-
نظری ندارم-

- .اجتناب از گردهمایی های اجتماعی از راه حل موثر برای کاهش شیوع کرونا است
- .تعطیلی گزینشی اماکن عمومی از راه حل موثر برای کاهش شیوع کرونا است
- .بکارگیری اصول بهداشت دست از راه حل موثر برای کاهش شیوع کرونا است

- عمل کردن به اصول تنفسی (رعایت فاصله هنگام صحبت، استفاده از دستمال یا آرنج دست جلوی دهان حین عطسه و سرفه) از راه حل موثر برای کاهش شیوع کرونا است.
- قرنطینه ی کامل اجتماعی از راه حل موثر برای کاهش شیوع کرونا است.

سوالات زیر راجع به جمعیت شناسی شرکت کنندگان در این پژوهش میباشد.

Q5.

جنسیت:

- مرد-
- زن-

Q6.

سن:

- کمتر از ۲۱ سال
- سال ۲۱-۳۰
- سال ۳۱-۴۰
- سال ۴۱-۵۰
- سال ۵۱-۶۰
- سال ۶۱-۷۰
- سال ۷۱-۸۰
- بیشتر از ۸۰ سال

Q7.

کار روزانه ی شما جزو کدام یک از موارد زیر میباشد؟

- شاغل
- خانه دار
- دانش آموز / دانش جو
- بازنشسته
- بیکار
- غیره

Q8.

وضعیت خانوار

- مجرد
- پدر/مادر مجرد با فرزند
- متاهل بدون فرزند

- متاهل با فرزند
- (خانه اشتراکی (همخانه/هم اتاق
- غیره

Q9.
میزان تحسیلات

- دبستان/ راهنمایی
- دیپلم متوسطه
- تحسیلات دانشگاهی

Q10.
؟آیا شما عضو کادر درمانی (پزشک، پرستار یا دانشجوی علوم پزشکی) هستید

- بله
- خیر

Q11.
:کشور محل اقامت

Q12.
به عنوان مثال: بیماریهای تنفسی/ قلبی، اختلالات متابولیک مانند دیابت، پیشینه ی سرطان) ؟آیا از بیماری مزمن یا وضعیت نامناسب جسمی رنج میبرید (یا بیماریهایی که نیاز به درمان بلند مدت دارند

- بله
- خیر

Q13.
؟از چه طریقی به این پرسشنامه دسترسی یافتید

- فیسبوک
- توییتر
- اینستاگرام
- لینکد این
- واتس اپ
- غیره
