# Supplementary material for: Public perspectives on protective measures during the COVID-19 pandemic in the Netherlands, Germany and Italy: A survey study
Source: PLoS One. 2020 Aug 5;15(8):e0236917. doi: 10.1371/journal.pone.0236917 (PMC7406072; doi:10.1371/journal.pone.0236917)
Supplement: S2 Appendix — (PDF) [file pone.0236917.s003.pdf]

S2 Appendix 2. Daily stage classification of community containment measures taken by country in March 2020.

	Netherlands	Germany	Italy
1-3-2020	STAGE I	UNCLEAR	STAGE III
2-3-2020	STAGE I	UNCLEAR	STAGE III
3-3-2020	STAGE I	UNCLEAR	STAGE III
4-3-2020	STAGE I	UNCLEAR	STAGE III
5-3-2020	STAGE I	UNCLEAR	STAGE III
6-3-2020	STAGE I	UNCLEAR	STAGE III
7-3-2020	STAGE I	UNCLEAR	STAGE III
8-3-2020	STAGE I	UNCLEAR	STAGE III
9-3-2020	STAGE I	STAGE II	STAGE IV
10-3-2020	STAGE II	STAGE II	STAGE IV
11-3-2020	STAGE II	STAGE II	STAGE IV
12-3-2020	STAGE II	STAGE II	STAGE IV
13-3-2020	STAGE II	STAGE II	STAGE IV
14-3-2020	STAGE II	STAGE II	STAGE IV
15-3-2020	STAGE III	STAGE II	STAGE IV
16-3-2020	STAGE III	STAGE III	STAGE IV
17-3-2020	STAGE III	STAGE III	STAGE IV
18-3-2020	STAGE III	STAGE III	STAGE IV
19-3-2020	STAGE III	STAGE III	STAGE IV
20-3-2020	STAGE III	STAGE III	STAGE IV
21-3-2020	STAGE III	STAGE III	STAGE IV
22-3-2020	STAGE III	STAGE III	STAGE IV
23-3-2020	STAGE III	STAGE III	STAGE IV
24-3-2020	STAGE III	STAGE III	STAGE IV
25-3-2020	STAGE III	STAGE III	STAGE IV
26-3-2020	STAGE III	STAGE III	STAGE IV
27-3-2020	STAGE III	STAGE III	STAGE IV
28-3-2020	STAGE III	STAGE III	STAGE IV
29-3-2020	STAGE III	STAGE III	STAGE IV
30-3-2020	STAGE III	STAGE III	STAGE IV
31-3-2020	STAGE III	STAGE III	STAGE IV

Stage I: Low Impact Containment Measures (corresponding to interventions 1,2, and 3)
Stage II: Focused Measures to Increase Social Distance (corresponding to interventions 4 and 5)
Stage III: Community-Wide Measures to Increase Social Distance (corresponding to intervention 6)
Stage IV: Widespread Community Quarantine, Including Cordon Sanitaire (corresponding to intervention 7)

Interventions of Community Containment Measures, Centers for Disease Control and Prevention.

As adopted from https://www.cdc.gov/sars/guidance/d-quarantine/app1.html.

- **Intervention 1:** Passive monitoring
- **Intervention 2:** Active Monitoring without Explicit Activity Restrictions
- **Intervention 3:** Active Monitoring with Activity Restrictions (Quarantine)
- **Intervention 4:** Working Quarantine
- **Intervention 5:** Focused Measures to Increase Social Distance
- **Intervention 6:** Community-Wide Measures to Increase Social Distance
- **Intervention 7:** Widespread Community Quarantine, Including Cordon Sanitaire

Daily stage classification in timelines: the Netherlands

Date	Government	Action
27.01.2020	National	The Ministry of Health Care and Sports announces that COVID-19 is classified as an illness of category "A": residents have a reporting obligation when suspicion arises that they are infected, or they suspect others to be infected.
02.02.2020	National	The Ministry of Foreign Affairs brings Dutch citizens back from Wuhan. None of the 15 evacuated people are infected.
05.03.2020	National	Minister Bruins announces that the national organization of the GGD (local public health service) is going to distribute face masks and other protective gear among all doctors and health care providers in the Netherlands.
09.03.2020	National	Jaap van Dissel (virologist and head of the RIVM) and the government ask residents to follow the advice closely and stop shaking hands.<br><br>Residents of the province of Noord-Brabant are asked to work from home as much as possible.
10.03.2020	National	Family members of someone infected with SARS-CoV-2 will not be tested anymore, in order to save the number of available tests and to relieve test laboratories.
	National	Big events in Noord-Brabant are canceled (festivals, concerts, Carnaval, fair at the Technical University of Eindhoven, football games). Schools, restaurants, cafes, and clubs are still open.
12.03.2020	National	Minister Bruins announces new measures, at least until 31.03.2020. Residents who have minor complaints (having a cold, coughing, with or without fever) should stay home.<br><br>Meetings with 100 people or more are not allowed. Residents from all regions in the Netherlands should work from home as much as possible. Vulnerable citizens should avoid large groups and public transport and visiting vulnerable people should be limited. Health care providers and residents who do vital jobs are asked to only stay home if they have complaints along with a fever.<br><br>Colleges and universities are advised to provide online education. Primary schools, secondary schools and vocational schools (MBO) remain open for now. Later that evening a motion is accepted in the "Tweede Kamer" (House of Representatives): parents who keep their healthy children at home will not receive a fine.
13.03.2020	National	Hospitals throughout the Netherlands postpone non-urgent operations, partly because of the impending shortage of protective gear (such as mouth masks), and to ensure staff is available for acute care. The number of Intensive Care (IC) beds is being expanded.
15.03.2020	National	Minister Bruins and minister Slob announce new, sharper measures, at least until 06.04.2020. As of 6 pm this day, all catering industries will be closed (restaurants, cafes, coffee shops, sex clubs, other places to go out), sport clubs and saunas. Delivery of meals and beverages is still allowed.<br><br>All schools in the Netherlands are closed and are asked to prioritise adapted education for graduating students. Special shelter at schools will be opened for children from parents working in "vital jobs" (such as healthcare, education, public transport, food supply, transport, waste processing, day-care, media and communication, emergency services, and necessary

		government processes).<br><br>All residents should keep 1.5 meters distance from each other, when possible.
16.03.2020	National	In addition to the measures of 15.03.2020: take-away of meals and beverages and take-away from coffee shops (Dutch establishments for the sale of cannabis) are also allowed.
	National	Speech from Prime Minister Mark Rutte. The Cabinet pursues group-immunity, since the expectation will be that most of the residents in the Netherlands will get infected with the coronavirus.
17.03.2020	National	Ministers Hoekstra, Koolmees and Wiebes announce a support package, containing 10-20 billion euros for the first 3 months. There will be support for reduction of working hours (the government will take over 90% of the salaries), self-employed residents, and temporarily no payment of tourist taxes. Companies will not have to pay taxes in the coming 3 months, there is a postponement of 6 months of loan repayment from small businesses, and the companies that are most affected will immediately receive €4.000 for support.
18.03.2020	National	The Eurovision Song Contest 2020 in Rotterdam has officially been canceled.
19.03.2020	National	Minister Bruins resigns, after he fainted because of overtiredness. Minister van Rijn will temporarily replace him, and he will be assisted by minister De Jonge during this COVID-19epidemic.
20.03.2020	National	Speech from king Willem-Alexander, to encourage the residents of the Netherlands in these times. He speaks of alertness, solidarity and warmth: we should all try to hold on to these things, which will help us through this crisis, even if it takes longer than expected.
23.03.2020	National	Existing measures will be expanded and new measures will be added. People are only allowed to go outside if absolutely necessary like for grocery shopping or for work if employed in vital jobs. If outside, a distance of 1.5 meter must be kept, no social gatherings of groups are allowed. Stay home if anyone in the household shows signs or symptoms of corona.<br><br>All social gatherings are prohibited until 1.6.2020, with an exception of funerals and church weddings. Shops and public transport are obliged to keep distance.<br><br>Mayors are allowed to prohibit grouping of people in public areas, if groups larger than 3 people.<br><br>van Rijn. There are new measures and current measures are expanded: residents should stay home as much as possible. People should only go outside for work, if they cannot work from home, for groceries or to take care of other people. Residents are allowed to get a breath of fresh air, but not in groups. Everyone should always keep a minimum of 1.5 meters distance from each other, avoid social gatherings and groups.<br><br>At home it is also advised to have a maximum of 3 people visiting during which everyone should keep their distance too. If someone in a household has fever, every member of the household has to stay home. The exception are people who have vital jobs, unless they fall ill themselves.<br><br>All social gatherings are prohibited until 01.06.2020, regardless of the number of people, There will be a special exception on this rule for funerals and church weddings.<br>Shops and public transport are obligated to take measures to keep people at distance, such as a door policy. Hairdressers, beauticians, and other contact professions in the field of beauty and

		grooming are not allowed to practice their profession until at least 06.04.2020.<br><br>Mayors are allowed to prohibit grouping of people in certain areas, such as parks, beaches or districts. This applies to groups of 3 or more people, who are not able to keep a distance of 1.5 meters from each other. Exceptions are people who live in the same household and children. Markets are still allowed if they are an essential part of the food chain. Mayors will have the possibility to act faster and easier to maintain the measures, through an emergency ordinance. Fines can also be imposed, if measures are not being followed.
24.03.2020	National	This year, there will not be any national, centrally organized exams on secondary schools. Instead, graduation classes will be able to achieve high school diplomas based on the locally organised school exams.
27.03.2020	National	Prime Minister Rutte holds another press conference. Again, he asks residents to follow the previously mentioned advice closely: stay at home as much as possible, keep at least 1.5 meter distance from each other and stay home if there are any complaints. He also specifically asked people to stay home on the weekend, since there were still a lot of people outside in the previous weekend. Furthermore, he repeated his amazement of residents who work in vital processes (such als people in health care, but also police officers, cleaners, and people working in logistics and transport). On 31.03.2020 a meeting will be held about how measures will continue after 06.04.2020, the so-called ""expire""-date of the current measures. Eventually, he also speaks about the impact on the economy, assures residents that the government is trying to limit the impact as much as possible, and that there will be economic support for several people in specific situations.
31.03.2020	National	Prime Minister Rutte states in a press conference that governmental measures will be extended until April 28th.

Daily stage classification in timelines: Germany

Date	Government	Action
09.03.2020	Federal	Agreement to facilitate a fund to compensate reduced hours, infrastructure investment, liquidity support, financial guarantees, and tax deferrals for business affected by the COVID-19 epidemic
	Federal	Minister of Health Spahn urges the population to "do their part": washing hands, keeping distance, refraining from attending public events and traveling, working from home. He recommends canceling events with more than 1000 people, but does not call for closure of schools and daycare.
	Bavaria	Consideration to cancel all events with more than 1000 people until April 10th, following Health Minister Spahn's recommendation.
10.03.2020	Federal	Spahn urges the population to act in solidarity and to brace for further restrictions of public life, in order to lower the number of new infections. Minister for Economic Affairs Peter Altmaier announces expansion of governmental support if necessary and expects a recession.
11.03.2020	Federal	Angela Merkel makes first public statements on the coronavirus crisis: containing the spread of the virus is the central task; we need to slow it down so we don't stress our healthcare system too much; the situation is similar to 2008 with regards to its severity, but different regarding the number of unknown variables.
12.03.2020	Federal	President Steinmeier calls for solidarity among the population
	Federal	Minister for Family Affairs, Senior Citizens, Women and Youth Giffey asks for specific protection for the elderly
	All states	Prime Ministers agree to "further contain the spread of the virus". No other agreements on coordinated action
	Federal	Chancellor Merkel reports that she and the state Prime Ministers agreed to cancel all non-essential events with less than 1000 people. She reassures that the German government will do whatever is necessary to stabilize the economy, regardless of new debts, and urges to avoid any social contact that isn't necessary, but leaves closure of schools and daycare to the individual states.
	Bavaria	Prime Minister Markus Söder no longer rules out state-wide closure of schools and daycare. Decision to be made on March 12th or 13th
13.03.2020	Federal	Minister for Finance and Vice Chancellor Scholz calls for optimism: "we will master this crisis together". He expects that many businesses will have to reduce their production capacities.
	Federal	Parliament passes law to facilitate short-time work
	Federal	Scholz promises unlimited loans to help businesses

	Federal	Merkel announces that the German government will do whatever it takes to get through this "unique crisis"
	Saarland	Starting on March 16th: closure of all public schools and daycare until April 24th (end of Easter holidays); limitations on visits to hospitals and retirement homes.
	Berlin	Starting on March 16th: closure of all public schools and daycare.
	Bavaria	Cafes and Restaurants will not be closed, recommendation to cancel events with more than 100 people"
14.03.2020	Federal	Spahn urges German travelers returning from Austria, Italy, and Switzerland to "avoid any unnecessary contacts with others" and to "self-quarantine" for two weeks
	Federal	Minister of Food, Agriculture and Consumer Protection Klöckner asks citizens not to refrain from panic buying but to fill their supplies moderately. Supply shortfalls do currently not exist in Germany
	Berlin	Starting on March 15th: prohibition of events with more than 50 people,closure of cinemas, theatres, museums, concert halls, clubs, bars, trade shows, and arcades.
	Mecklenburg-Western Pomerania	Starting on March 16th: closure of all public schools and daycare
15.03.2020	Federal	Minister of Defence Kramp-Karrenbauer pledges the German military's full support to fight the coronavirus crisis
	Federal	Starting on March 16th, 8:00 AM: partial closure of borders to Austria, France, Switzerland and Danish border
	Federal	Giffey promised more support for short-time employees with children, mainly to give them access to child benefits.
	Hamburg	Prohibition of all public and private events regardless of the number of people
16.03.2020	Federal	Head of the Chancellery Braun assures that there are no supply shortages
	Federal	Ministry for Economic Affairs and Energy expects a significant and prolonged economic downturn
	Federal	Members of the German parliament discuss if constitutional changes are necessary to maintain legislative powers during crises
	Federal & all states	Common agreement: closure of shops except for grocery stores,<br>drug stores, banks, petrol stations, and other essential businesses,<br>limited opening hours for restaurants, closure of playgrounds, hotels and other accommodations are closed for tourists, prohibition of religious services and gatherings, association meetings, and bus travel
	Federal	Minister of Justice and Consumer Protection Lambrecht announces that businesses that can't pay their bills due to the coronavirus crisis are exempt from filing for bankruptcy until September

	Federal	Merkel calls the coronavirus crisis the biggest challenge since World War II. Besides the decisions made earlier today, holiday travel within Germany and abroad will be prohibited temporarily
	Bavaria	Announcement of a state of emergency
17.03.2020	Federal	Robert-Koch-Institut sets risk assessment to "High"
	Federal	Robert-Koch-Institut: restrictions on public life could last for 2 years
	Federal & all states	Agreement on hospital emergency plan: creation of temporary hospital capacities in hotels, public halls, etc. in order to provide more space for intensive care units (ICUs) in hospitals
	Schleswig-Holstein	Starting on March 18th: tourists are no longer allowed to enter the state
18.03.2020	Federal	Braun: currently no plans for nationwide curfew, however, social contacts must be reduced significantly to slow down spread of the virus
	Federal	Minister for Education and Research Karliczek stresses that<br>development of a vaccine takes time, no quick results to be expected
	Federal	Angela Merkel will address the public in a TV speech
19.03.2020	Saxony-Anhalt	Closure of hotels, camping grounds, and bed & breakfasts for tourists until April 19th
20.03.2020	Regional	In Rhineland-Palatinate, Bremen, Hesse, Baden-Württemberg, Lower Saxony, Hamburg, Mecklenburg-Western Pomerania:<br>Closure of restaurants, prohibition of meetings with more than 3-5 people (number can differ per state)
21.03.2020	Federal	Governments to take on €150 billion in new debt
	Federal & all states	Meeting on potential nationwide curfews planned for Sunday
	Berlin	Closure of restaurants, prohibition of meetings with more than 10 people
22.03.2020	Federal	Scholz is skeptical of curfews
	Federal	Minister for Transport, Building and Urban Development Scheuer considers plans to keep logistics hubs open 24 hours to prevent potential supply shortages
	Federal	Government increases aid to hospitals to €7.8 billion after criticism
	Federal	Nationwide 2-week prohibition of meetings with more than 2 people, except for core families, partners, job reasons, public transit, and funerals, with fines up to €25,000; closure of all remaining restaurants and hair dressers
23.03.2020	Federal	Cabinet provide for financial aid package today, including up to €50 billion direct support for small businesses and freelancers, facilitation of government investment in large

		companies, temporary prohibition of evictions due to rent debt, and easier access to short-time work compensation
	Federal	Scholz: based on estimations of a 5%-drop in economic growth, the government passed a financial aid package of €750 billion, including €156 billion new debt
24.03.2020	Northrine-Westphalia	Fines for not following curfew rules: Meeting outside with more than 2 people: €200 per person, having a picnic or barbecue: €250 per person, organising a sports event: €1000, eating take-out closer than 50m from restaurant: €200, violating visiting rules in retirement homes: €200, keeping a bar, club or gym open: €5000, keeping a restaurant open: €4000
	Brandenburg	Cancellation of larger events in May and June
25.03.2020	Federal	Giffey is afraid of increased cases of domestic violence due to restrictions
	Federal	Scholtz stresses the need to work together to ensure three things: keep the health system working, protect people's incomes and protect jobs & businesses.
	All states	Ministers for Education agree not to cancel school-leaving exams
	All states	The Bundesrat (the states' representative body at the federal level) agrees to a supplementary budget without any amendments
26.03.2020	Saarland	State tightens movement restrictions
	Federal	Annegret Kramp-Karrenbauer calls for isolation of risk groups
27.03.2020	Federal	Angela Merkels warns that debate on how to end restrictions comes too early
28.03.2020	Federal	Helge Braun clarifies that there will be no loosening of restrictions before April 20, senior citizens will have to live with restrictions even longer
29.03.2020	Federal	Olaf Scholz is against loosening restrictions for economic reasons. Corona Premiums for employees up to €1500 are exempt from taxes. Peter Altmaier calls for a comprehensive aid program for German economy to make it more competitive as part of measures to fight Corona.
30.03.2020	Federal & all states	Government sees currently no room for loosening restrictions any time soon. State prohibits any further admissions to retirement homes. Also, in all states the restrictions are extended until April 19, 2020. Angela Merkels third test is also negative.

Daily stage classification in timelines: Italy

Date	Government	Action
31.01.2020	National	Government suspends all flights to and from China and declares 'a state of emergency'. There was also the introduction of thermal scanners and temperatures of all international passengers have to be checked at Italian airports.
22.02.2020	National	Quarantine zones (red zones) are established in more than 11 municipalities in northern Italy. The surrounding areas are yellow zones. Non-compliance to the measures can be punished (from a fine of €206,- to imprisonment for up to 3 months)<br><br>Several activities, such as the Carnival of Venice, are cancelled.<br><br>Several universities in Lombardy, Veneto, Trentino Alto-Adige, Piedmont and Emilia-Romagna cancel all activities.
26.02.2020	National	Locatelli (Director of the Italian National Institute of Health) declares that testing will only be done on symptomatic patients.
27.02.2020	National	In Taranto, Apulia, all schools and several universities are closed until the 29th of February. Online programmes are being developed to teach students online, which will start around the 2nd of March.
28.02.2020	National	Universities in Lombardy extend the closures until March 7.
01.03.2020	National	President Conte signed a decree, thereby incorporating and extending some of the measures already adopted for the containment and management of the COVID-19 epidemic. The decree introduces further measures aimed at regulating the interventions in a unified way and ensuring uniformity throughout the nation.<br><br>Italy is divided into 3 areas: 1. Red zone (Bertonico, Casalpusterlengo, Castelgerundo, Castiglione D'Adda, Codogno, Fombio, Maleo, San Fiorano, Somaglia and Terranova dei Passerini in Lombardy, and the municipality of Vò in Veneto). Here the whole population is quarantined. 2. Yellow zone (regions of Lombardy, Veneto and Emilia-Romagna). Social and sports events are being canceled and schools, theatres, clubs and cinemas are closed. 3. The rest of the country: safety and prevention measures are displayed in public places, and special cleansings of public transport are performed.
04.03.2020	National	President Conte and the Minister of Education, Lucia Azzolina, announced suspension throughout Italy for educational activities (e.g. schools and universities) from 5 to 15 March in order to contain the epidemic. Italy has reached 100 deaths.
06.03.2020	National	The Council of Ministers approved a decree-law that introduces extraordinary and urgent measures to counter the epidemiological emergency from COVID-19.
08.03.2020	National	Prime Minister Conte has signed a new decree implementing further measures for the national containment and management of the epidemiological emergency from COVID-19, illustrated in a press conference.<br><br>More specifically, the new decree expands the lockdown to the whole region of Lombardy and 14 other northern provinces. All gyms, swimming pools and wellness centers are shut and

		shopping malls have to be closed for the weekend. Other commercial activities can remain open, as long as a distance of at least 1 meter can be assured.
09.03.2020	National	All sports events in Italy are cancelled, except for Italian sports clubs or national teams that participate in international competitions. It is also forbidden to gather people in public places or locations open to the public. These provisions have effect from March 10th, 2020 and are effective until April 3rd, 2020. In the evening, the President illustrated the decree at a press conference.
11.03.2020	National	In the evening, President Conte signed the new decree containing further measures. During the live press conference, the President announced the closure of all commercial and retail activities, with the exception of grocery stores, basic necessities, pharmacies and parapharmacies.All commercial and retail businesses, except for essential services (such as supermarkets and pharmacies), are closed.
19.03.2020	National	A task force of doctors from all over Italy is created to support regional and national healthcare structures. The task force will consist of a maximum of 300 doctors that participate on a voluntary basis. Thereby, the army is deployed in Bergamo, the city most affected by COVID-19, as the city itself can no longer cope with the number of deaths.
20.03.2020	National	The Ministry of Health signed an decree for stricter regulation of unrestricted movement, such as public access to parks, villas, play areas and public gardens, and restrictions on recreational or outdoor activities, such as open-air sports and running. Movement towards a residence different from one’s main residence is prohibited. The ordinance also requires the closure of the food and beverage service establishments, located inside the railway and lake stations, and local service and refueling areas.
22.03.2020	National	A new ordinance is adopted which prohibits all persons from moving or moving with public or private means of transport in a municipality other than that in which they are located, except for essential work, of absolute urgency or for health reasons.<br><br>In the evening the President Conte signed a decree for the closure of non-essential or strategic production activities, such as grocery stores, pharmacies, basic necessities shops and essential services. The provisions have effect from March 23th, 2020 and are effective until April 3rd, 2020. The same decree applies, to those referred to in the Prime Minister's decree of March 11th, 2020, as well as to those provided for by the Minister of Health of March 20th, 2020.
24.03.2020	National	The Council of Ministers approves a decree-law, which makes it easier for regional governments to bring in stricter rules for predetermined periods (lasting no more than thirty days) until the end of the state of emergency, set at 31 July 2020. The application of the measures may be modulated up or down according to the epidemiological trend of COVID-19. Prime minister Conte announces bigger fines when rules around the quarantine are being broken, with fines up to €3.000,-.
25.03.2020	National	Companies that had not been suspended by the Prime Ministerial Decree of 22 March 2020 and who, due to the effect of this decree, will have to suspend their business, will be allowed to complete the activities necessary for the suspension, including the shipment of goods in storage, up to the date March 28, 2020.
30.03.2020	National	The government informed its citizens that they should be ready for a lengthy confinement that would only be lifted gradually. Italy has nearly 10,800 deaths due to COVID-19, the highest death toll among European countries.
