# Supplementary material for: Public perspectives on protective measures during the COVID-19 pandemic in the Netherlands, Germany and Italy: A survey study
Source: PLoS One. 2020 Aug 5;15(8):e0236917. doi: 10.1371/journal.pone.0236917 (PMC7406072; doi:10.1371/journal.pone.0236917)
Supplement: S3 Appendix — (PDF) [file pone.0236917.s004.pdf]

S3 Appendix 3.

Table a: Belief in the effectiveness of policy recommendations during the COVID-19 pandemic in the Netherlands, by gender.

	Male				Female				Other			
No.	2,492				6,095				24			
**Belief in effectiveness of recommendations (%)**	Probably true	Probably false	Don't know	Missing	Probably true	Probably false	Don't know	Missing	Probably true	Probably false	Don't know	Missing
Avoid social gatherings	2,447 (98.7)	27 (1.1)	6 (0.2)	12	6,008 (99.0)	33 (0.5)	25 (0.4)	29	24 (100.0)	0 (0.0)	0 (0.0)	0
Selective closure of public places/locations	2,368 (95.4)	78 (3.1)	36 (1.5)	10	5,767 (95.2)	156 (2.6)	135 (2.2)	37	23 (95.8)	0 (0.0)	1 (4.2)	0
Implementation of hand hygiene measures	2,334 (93.8)	86 (3.5)	68 (2.7)	4	5,885 (97.0)	81 (1.3)	100 (1.7)	29	21 (87.5)	2 (8.3)	1 (4.2)	0
Implementation of respiratory measures	2,338 (94.3)	87 (3.5)	55 (2.2)	12	5,779 (95.4)	141 (2.3)	141 (2.3)	34	22 (91.7)	2 (8.3)	0 (0.0)	0
Complete social lockdown/isolation	1,519 (61.3)	654 (26.4)	306 (12.3)	13	3,532 (58.4)	1,322 (21.8)	1,190 (19.7)	51	12 (52.2)	7 (30.4)	4 (17.4)	1

For full questionnaire and wording, see Supplement I.
Response percentages may not add up to 100% due to rounding.

Table b: Belief in the effectiveness of policy recommendations during the COVID-19 pandemic in the Netherlands, by age.

	≤ 20 years				21-40 years				41-60 years				> 60 years			
No.	675				4,034				2,836				1,066			
**Belief in effectiveness of recommendations (%)**	Probably true	Probably false	Don't know	Missing	Probably true	Probably false	Don't know	Missing	Probably true	Probably false	Don't know	Missing	Probably true	Probably false	Don't know	Missing
Avoid social gatherings	658 (97.8)	10 (1.5)	5 (0.7)	2	3,982 (99.0)	27 (0.7)	12 (0.3)	13	2,791 (99.0)	16 (0.6)	11 (0.4)	18	1,048 (99.0)	7 (0.7)	3 (0.3)	8
Selective closure of public places/locations	630 (93.6)	26 (3.9)	17 (2.5)	2	3,803 (94.5)	132 (3.3)	88 (2.2)	11	2,702 (96.2)	57 (2.0)	49 (1.8)	28	1,023 (96.5)	19 (1.8)	18 (1.7)	6
Implementation of hand hygiene measures	628 (93.3)	23 (3.4)	22 (3.3)	2	3,837 (95.3)	101 (2.5)	87 (2.2)	9	2,738 (97.0)	37 (1.3)	46 (1.6)	15	1,037 (97.9)	8 (0.8)	14 (1.3)	7
Implementation of respiratory measures	591 (88.0)	42 (6.3)	39 (5.8)	3	3,777 (94.1)	135 (3.4)	103 (2.6)	19	2,742 (97.1)	37 (1.3)	43 (1.5)	14	1,029 (97.4)	16 (1.5)	11 (1.0)	10
Complete social lockdown/isolation	318 (47.5)	208 (31.1)	143 (21.4)	6	2,511 (62.7)	924 (23.1)	573 (14.3)	26	1,660 (59.0)	613 (21.8)	541 (19.2)	22	574 (54.4)	238 (22.6)	243 (23.0)	11

For full questionnaire and wording, see Supplement I.
Response percentages may not add up to 100% due to rounding.

Table c: Belief in the effectiveness of policy recommendations during the COVID-19 pandemic in the Netherlands, by daily activity.

	Paid Job				Homemaker/ Unemployed				Student				Retired				Other			
No.	4,885				573				2,272				557				324			
**Belief in effectiveness of recommendations (%)**	Probably true	Probably false	Don't know	Missing	Probably true	Probably false	Don't know	Missing	Probably true	Probably false	Don't know	Missing	Probably true	Probably false	Don't know	Missing	Probably true	Probably false	Don't know	Missing
Avoid social gatherings	4,813 (99.0)	31 (0.6)	16 (0.3)	25	567 (99.5)	2 (0.4)	1 (0.2)	3	2,233 (98.6)	23 (1.0)	9 (0.4)	7	550 (99.5)	2 (0.4)	1 (0.2)	4	316 (98.1)	2 (0.6)	4 (1.2)	2
Selective closure of public places/ locations	4,635 (95.5)	135 (2.8)	84 (1.7)	31	549 (96.3)	8 (1.4)	13 (2.3)	3	2,138 (94.3)	72 (3.2)	57 (2.5)	5	537 (97.3)	9 (1.6)	6 (1.1)	5	299 (93.2)	10 (3.1)	12 (3.7)	3
Implementation of hand hygiene measures	4,693 (96.5)	87 (1.8)	84 (1.7)	21	551 (96.3)	8 (1.4)	13 (2.3)	1	2,144 (94.5)	64 (2.8)	60 (2.7)	4	542 (98.4)	4 (0.7)	5 (0.9)	6	310 (96.0)	6 (1.9)	7 (2.2)	1
Implementation of respiratory measures	4,678 (96.2)	102 (2.1)	82 (1.7)	23	536 (94.4)	15 (2.6)	17 (3.0)	5	2,078 (91.8)	100 (4.4)	86 (3.8)	8	540 (98.5)	7 (1.3)	1 (0.2)	9	307 (95.1)	6 (1.9)	10 (3.1)	1
Complete social lockdown/isolation	2,932 (60.5)	1,114 (23.0)	801 (16.5)	38	368 (64.7)	89 (15.6)	112 (19.7)	4	1,267 (56.1)	594 (26.3)	398 (17.6)	13	304 (55.4)	123 (22.4)	122 (22.2)	8	192 (59.6)	63 (19.6)	67 (20.8)	2

For full questionnaire and wording, see Supplement I.
Response percentages may not add up to 100% due to rounding.

Table d: Belief in the effectiveness of policy recommendations during the COVID-19 pandemic in the Netherlands, by household composition.

	Single				Parent(s) with child(ren)				Living with partner				Shared flat				Other			
No.	1,391				2,740				2,339				1,558				583			
**Belief in effectiveness of recommendations (%)**	Probably true	Probably false	Don't know	Missing	Probably true	Probably false	Don't know	Missing	Probably true	Probably false	Don't know	Missing	Probably true	Probably false	Don't know	Missing	Probably true	Probably false	Don't know	Missing
Avoid social gatherings	1,368 (98.6)	11 (0.8)	8 (0.6)	4	2,696 (99.1)	15 (0.6)	9 (0.3)	20	2,313 (99.2)	14 (0.6)	4 (0.2)	8	1,537 (99.0)	11 (0.7)	4 (0.3)	6	565 (97.4)	9 (1.6)	6 (1.0)	3
Selective closure of public places/locations	1,311 (94.8)	42 (3.0)	30 (2.2)	8	2,609 (96.1)	64 (2.4)	41 (1.5)	26	2,229 (95.6)	57 (2.5)	45 (1.9)	8	1,477 (94.9)	48 (3.1)	32 (2.1)	1	532 (91.9)	23 (4.0)	24 (4.2)	4
Implementation of hand hygiene measures	1,322 (95.5)	35 (2.5)	27 (2.0)	7	2,632 (96.6)	40 (1.5)	53 (1.9)	15	2,278 (97.7)	30 (1.3)	23 (1.0)	8	1,475 (94.7)	42 (2.7)	40 (2.6)	1	533 (91.7)	22 (3.8)	26 (4.5)	2
Implementation of respiratory measures	1,312 (95.0)	40 (2.9)	29 (2.1)	10	2,620 (96.4)	50 (1.8)	49 (1.8)	21	2,250 (96.5)	43 (1.8)	38 (1.6)	8	1,446 (93.1)	59 (3.8)	48 (3.1)	5	511 (88.0)	38 (6.5)	32 (5.5)	2
Complete social lockdown/isolation	860 (62.5)	302 (21.9)	215 (15.6)	14	1,662 (61.1)	573 (21.1)	485 (17.8)	20	1,377 (59.3)	507 (21.8)	438 (18.9)	17	886 (57.2)	427 (27.6)	235 (15.2)	10	278 (48.0)	174 (30.1)	127 (22.0)	4

For full questionnaire and wording, see Supplement I.
Response percentages may not add up to 100% due to rounding.

Table e: Belief in the effectiveness of policy recommendations during the COVID-19 pandemic in the Netherlands, by highest educational qualification.

	**Primary/Secondary**				**Tertiary vocational**				**Tertiary academic**			
No.	635				1,991				5,985			
**Belief in effectiveness of recommendations (%)**	Probably true	Probably false	Don't know	Missing	Probably true	Probably false	Don't know	Missing	Probably true	Probably false	Don't know	Missing
Avoid social gatherings	616 (98.1)	8 (1.3)	4 (0.6)	7	1,952 (98.6)	19 (1.0)	9 (0.5)	11	5,911 (99.1)	33 (0.6)	18 (0.3)	23
Selective closure of public places/locations	587 (93.6)	21 (3.4)	19 (3.0)	8	1,884 (95.3)	58 (2.9)	35 (1.8)	14	5,687 (95.4)	155 (2.6)	118 (2.0)	25
Implementation of hand hygiene measures	606 (96.2)	6 (1.0)	18 (2.9)	5	1,894 (95.7)	48 (2.4)	37 (1.9)	12	5,740 (96.2)	115 (1.9)	114 (1.9)	16
Implementation of respiratory measures	588 (93.6)	15 (2.4)	25 (4.0)	7	1,870 (94.5)	64 (3.2)	45 (2.3)	12	5,681 (95.4)	151 (2.5)	126 (2.1)	27
Complete social lockdown/isolation	343 (54.6)	137 (21.8)	148 (23.6)	7	1,091 (55.2)	465 (23.5)	419 (21.2)	16	3,629 (61.1)	1,381 (23.2)	933 (15.7)	42

For full questionnaire and wording, see Supplement I.
Response percentages may not add up to 100% due to rounding

Table f: Belief in the effectiveness of policy recommendations during the COVID-19 pandemic in the Netherlands, by healthcare provider or (bio-)medical student, and by chronic illness or being in poor medical condition.

	Healthcare provider or (bio-)medical student								Chronic illness or being in poor medical condition							
	Yes				No				Yes				No			
No.	1,572				7,039				7,083				1,528			
**Belief in effectiveness of recommendations (%)**	Probably true	Probably false	Don't know	Missing	Probably true	Probably false	Don't know	Missing	Probably true	Probably false	Don't know	Missing	Probably true	Probably false	Don't know	Missing
Avoid social gatherings	1,547 (98.9)	11 (0.7)	7 (0.5)	7	6,932 (99.0)	49 (0.7)	24 (0.3)	34	1,508 (99.2)	7 (0.5)	6 (0.4)	7	6,971 (98.9)	53 (0.8)	25 (0.4)	34
Selective closure of public places/locations	1,509 (96.2)	36 (2.3)	23 (1.5)	4	6,649 (95.0)	198 (2.8)	149 (2.1)	43	1,462 (96.3)	36 (2.4)	20 (1.3)	10	6,696 (95.0)	198 (2.8)	152 (2.2)	37
Implementation of hand hygiene measures	1,524 (97.3)	22 (1.4)	20 (1.3)	6	6,716 (95.8)	147 (2.1)	149 (2.1)	27	1,476 (97.0)	27 (1.8)	19 (1.3)	6	6,764 (95.9)	142 (2.0)	150 (2.1)	27
Implementation of respiratory measures	1,491 (95.2)	39 (2.5)	37 (2.4)	5	6,648 (95.0)	191 (2.7)	159 (2.3)	41	1,458 (96.2)	27 (1.8)	30 (2.0)	13	6,681 (94.8)	203 (2.9)	166 (2.4)	33
Complete social lockdown/isolation	946 (60.6)	379 (24.3)	235 (15.1)	12	4,117 (58.9)	1,604 (23.0)	1,265 (18.1)	53	955 (63.3)	292 (19.4)	262 (17.4)	19	4,108 (58.4)	1,691 (24.0)	1,238 (17.6)	46

For full questionnaire and wording, see Supplement I.
Response percentages may not add up to 100% due to rounding

Table g: Being informed about policy recommendations during the COVID-19 pandemic in the Netherlands, by gender.

	Male	Female	Other
No.	2,492	6,095	24
**Primary sources used to acquire information (%)***			
Television	1,823 (73.2)	4,773 (78.3)	17 (70.8)
Newspaper, mobile news application	1,685 (67.6)	3,725 (61.1)	12 (50.0)
Social media	970 (38.9)	2,457 (40.3)	14 (58.33)
Radio	346 (13.9)	727 (11.9)	4 (16.7)
Official health hotlines	37 (1.5)	90 (1.5)	0 (0.0)
Official health websites	896 (36.0)	2,456 (40.3)	9 (37.5)
Healthcare professionals	107 (4.3)	272 (4.5)	2 (8.3)
People I speak to on a daily basis	673 (27.0)	1,610 (26.4)	10 (41.7)
**Have been sufficiently informed (%)**			
Probably true	2,268 (91.0)	5,552 (91.1)	19 (79.2)
Probably false	87 (3.5)	183 (3.0)	1 (4.2)
Not sure	132 (5.3)	342 (5.6)	2 (8.3)
No opinion	5 (0.2)	18 (0.3)	2 (8.3)

For full questionnaire and wording, see Supplement I.
Response percentages may not add up to 100% due to rounding.
** Respondents were asked to choose the top 3 used information sources.*

Table h: Being informed about policy recommendations during the COVID-19 pandemic in the Netherlands, by age.

	≤ 20 years	21-40 years	41-60 years	> 60 years
No.	675	4,034	2,836	1,066
**Primary sources used to acquire information (%)***				
Television	502 (74.4)	2,803 (69.5)	2,363 (83.3)	945 (88.7)
Newspaper, mobile news application	385 (57.0)	2,698 (66.9)	1,686 (59. 5)	653 (61.3)
Social media	352 (52.2)	1,647 (40.8)	1,128 (39.8)	314 (29.5)
Radio	43 (6.4)	326 (8.1)	437 (15.4)	271 (25.4)
Official health hotlines	12 (1.8)	56 (1.4)	44 (1.6)	15 (1.4)
Official health websites	198 (29.3)	1,695 (42.0)	1,145 (40.4)	323 (30.3)
Healthcare professionals	24 (3.6)	201 (5.0)	124 (4.4)	32 (3.0)
People I speak to on a daily basis	334 (49.5)	1,349 (33.4)	467 (16.5)	143 (13.4)
**Have been sufficiently informed (%)**				
Probably true	587 (87.0)	3,605 (89.4)	2,635 (92.9)	1,012 (94.9)
Probably false	22 (3.3)	159 (3.9)	75 (2.6)	15 (1.4)
Not sure	62 (9.2)	259 (6.4)	117 (4.1)	38 (3.6)
No opinion	4 (0.6)	11 (0.3)	9 (0.3)	1 (0.1)

For full questionnaire and wording, see Supplement I.
Response percentages may not add up to 100% due to rounding.
** Respondents were asked to choose the top 3 used information sources.*

Table i: Being informed about policy recommendations during the COVID-19 pandemic in the Netherlands, by daily activity.

	Paid job	Homemaker/ Unemployed	Student	Retired	Other
No.	4,885	573	2,272	557	324
**Primary sources used to acquire information (%)***					
Television	3,822 (78.4)	467 (81.5)	1,588 (69.9)	502 (90.3)	234 (72.2)
Newspaper, mobile news application	3,094 (63.4)	308 (53.8)	1,500 (66.0)	348 (62.5)	172 (53.1)
Social media	1,846 (37.8)	268 (46.8)	1,008 (44.4)	167 (30.0)	152 (46.9)
Radio	700 (14.3)	76 (13.3)	112 (4.9)	146 (26.2)	43 (13.3)
Official health hotlines	69 (1.4)	7 (1.2)	37 (1.6)	5 (0.9)	9 (2.8)
Official health websites	2,003 (41.0)	208 (36.3)	858 (37.8)	154 (27.7)	138 (42.6)
Healthcare professionals	206 (4.2)	27 (4.7)	112 (4.9)	14 (2.5)	22 (6.8)
People I speak to on a daily basis	1,104 (22.6)	102 (17.8)	961 (42.3)	72 (12.9)	54 (16.7)
**Have been sufficiently informed (%)**					
Probably true	4,506 (92.2)	509 (88.8)	2,012 (88.6)	529 (95.0)	283 (87.4)
Probably false	146 (3.0)	20 (3.5)	85 (3.7)	9 (1.6)	11 (3.4)
Not sure	223 (4.6)	39 (6.8)	168 (7.4)	19 (3.4)	27 (8.3)
No opinion	10 (0.2)	5 (0.9)	7 (0.3)	0 (0.0)	3 (0.9)

For full questionnaire and wording, see Supplement I.
Response percentages may not add up to 100% due to rounding.
* Respondents were asked to choose the top 3 used information sources.

Table j: Being informed about policy recommendations during the COVID-19 pandemic in the Netherlands, by household composition.

	Single	Parent(s) with child(ren)	Living with partner	Shared flat	Other
No.	1,391	2,740	2,339	1,558	583
**Primary sources used to acquire information (%)***					
Television	978 (70.3)	2,221 (81.1)	1,865 (79.7)	1,091 (70.0)	458 (78.6)
Newspaper, mobile news application	855 (61.5)	1,652 (60.3)	1,529 (65.4)	1,060 (68.0)	326 (55.9)
Social media	582 (41.8)	1,108 (40.4)	812 (34.7)	657 (42.2)	282 (48.4)
Radio	212 (15.2)	336 (12.3)	396 (16.9)	72 (4.6)	61 (10.5)
Official health hotlines	19 (1.4)	39 (1.4)	30 (1.3)	30 (1.9)	9 (1.5)
Official health websites	557 (40.0)	1,129 (41.2)	895 (38.3)	568 (36.5)	212 (36.4)
Healthcare professionals	62 (4.5)	119 (4.3)	107 (4.6)	71 (4.6)	22 (3.8)
People I speak to on a daily basis	383 (27.5)	532 (19.4)	453 (19.4)	697 (44.7)	228 (39.1)
**Have been sufficiently informed (%)**					
Probably true	1,269 (91.2)	2,522 (92.0)	2,174 (93.0)	1,376 (88.3)	498 (85.4)
Probably false	46 (3.3)	77 (2.8)	58 (2.5)	68 (4.4)	22 (3.8)
Not sure	70 (5.0)	134 (4.9)	102 (4.4)	112 (7.2)	58 (10.0)
No opinion	6 (0.4)	7 (0.3)	5 (0.2)	2 (0.1)	5 (0.9)

For full questionnaire and wording, see Supplement I.
Response percentages may not add up to 100% due to rounding.
** Respondents were asked to choose the top 3 used information sources.*

Table k. Being informed about policy recommendations during the COVID-19 pandemic in the Netherlands, by highest educational qualification.

	Primary/Secondary	Tertiary vocational	Tertiary academic
No.	635	1,991	5,985
**Primary sources used to acquire information (%)***			
Television	502 (79.1)	1,684 (84.6)	4,427 (74.0)
Newspaper, mobile news application	285 (44.9)	1,035 (52.0)	4,102 (68.5)
Social media	292 (46.0)	872 (43.8)	2,277 (38.1)
Radio	106 (16.7)	317 (15.9)	654 (10.9)
Official health hotlines	15 (2.4)	24 (1.2)	88 (1.5)
Official health websites	183 (28.8)	688 (34.6)	2,490 (41.6)
Healthcare professionals	34 (5.4)	97 (4.9)	250 (4.2)
People I speak to on a daily basis	181 (28.5)	461 (23.2)	1,651 (27.6)
**Have been sufficiently informed (%)**			
Probably true	556 (87.6)	1,800 (90.4)	5,483 (91.6)
Probably false	27 (4.3)	69 (3.5)	175 (2.9)
Not sure	44 (6.9)	114 (5.7)	318 (5.3)
No opinion	8 (1.3)	8 (0.4)	9 (0.2)

For full questionnaire and wording, see Supplement I.
Response percentages may not add up to 100% due to rounding.
** Respondents were asked to choose the top 3 used information sources.*

Table I: Being informed about policy recommendations during the COVID-19 pandemic in the Netherlands, by healthcare provider or (bio-)medical student, and by chronic illness or being in poor medical condition.

	Healthcare provider or (bio-)medical student		Chronic illness or being in poor medical condition	
	Yes	No	Yes	No
No.	1,572	7,039	7,083	1,528
**Primary sources used to acquire information (%)***				
Television	1,199 (76.3)	5,414 (76.9)	1,230 (80.5)	5,383 (76.0)
Newspaper, mobile news application	903 (57.4)	4,519 (64.2)	894 (58.5)	4,528 (63.9)
Social media	590 (37.5)	2,851 (40.5)	611 (40.0)	2,830 (40.0)
Radio	154 (9.8)	923 (13.1)	224 (14.7)	853 (12.0)
Official health hotlines	41 (2.6)	86 (1.2)	22 (1.4)	105 (1.5)
Official health websites	712 (45.3)	2,649 (37.6)	608 (39.8)	2,753 (38.9)
Healthcare professionals	168 (10.7)	213 (3.0)	100 (6.5)	281 (4.0)
People I speak to on a daily basis	431 (27.4)	1,862 (26.5)	297 (19.4)	1,996 (28.2)
**Have been sufficiently informed (%)**				
Probably true	1,461 (92.9)	6,378 (90.6)	1,370 (89.7)	6,469 (91.3)
Probably false	34 (2.2)	237 (3.4)	51 (3.3)	220 (3.1)
Not sure	74 (4.7)	402 (5.7)	104 (6.8)	372 (5.3)
No opinion	3 (0.2)	22 (0.3)	3 (0.2)	22 (0.3)

For full questionnaire and wording, see Supplement I.
Response percentages may not add up to 100% due to rounding.
** Respondents were asked to choose the top 3 used information sources.*

Table m: Individual implementation of protective measures in response to COVID-19 pandemic in the Netherlands by gender.

	Male				Female				Other			
No.	2,492				6,095				24			
**Personal protective behaviors (%)**	**Y**	**N**	**NS**	**NA**	**Y**	**N**	**NS**	**NA**	**Y**	**N**	**NS**	**NA**
Cleaned or disinfected things you might touch more often than usual	955 (38.7)	1,307 (52.9)	207 (8.4)	23	3,115 (51.4)	2,435 (40.2)	506 (8.4)	39	13 (54.2)	11 (45.8)	0 (0.0)	0
Carried sanitizing hand gel with you when out and about	439 (17.9)	1,968 (80.2)	46 (1.9)	39	1,940 (32.5)	3,890 (65.2)	135 (2.3)	130	6 (31.6)	13 (68.4)	0 (0.0)	5
Used sanitizing hand gel to clean your hands, more often than usual	1,359 (55.3)	1,047 (42.6)	52 (2.1)	34	3,312 (55.3)	2,542 (42.5)	134 (2.2)	107	10 (45.5)	12 (54.6)	0 (0.0)	2
Reduced the amount you touch your eyes, nose and/or mouth	1,154 (46.4)	832 (33.5)	500 (20.1)	6	3,041 (50.0)	1,656 (27.3)	1,381 (22.7)	17	13 (54.2)	3 (12.5)	8 (33.3)	0
Followed a healthy diet or took vitamin supplements	1,155 (48.0)	1,141 (47.4)	113 (4.7)	83	3,380 (57.1)	2,278 (38.5)	257 (4.3)	180	13 (56.5)	10 (43.5)	0 (0.0)	1
Usually carried tissues with you when out and about	842 (34.5)	1,547 (63.4)	53 (2.2)	50	3,545 (59.8)	2,275 (38.4)	110 (1.9)	165	12 (52.2)	11 (47.8)	0 (0.0)	1
Usually used tissues when sneezing or coughing	1,024 (46.6)	1,095 (49.8)	81 (3.7)	292	3,504 (68.7)	1,447 (28.4)	151 (3.0)	993	14 (60.9)	9 (39.1)	0 (0.0)	1
Washed your hands with soap and water more often than usual	2,300 (92.4)	169 (6.8)	20 (0.8)	3	5,854 (96.1)	200 (3.3)	36 (0.6)	5	22 (91.7)	2 (8.3)	0 (0.0)	0
Kept child(ren) at home before any mandates were put in place	269 (49.4)	265 (48.6)	11 (2.0)	1,947	880 (55.1)	691 (43.2)	27 (1.7)	4,497	2 (66.7)	1 (33.3)	0 (0.0)	21
**Limiting interactions with people (%)**	**Y**	**N**	**NS**	**NA**	**Y**	**N**	**NS**	**NA**	**Y**	**N**	**NS**	**NA**
Cancelled or postponed a social event	2,256 (92.6)	122 (5.0)	59 (2.4)	55	5,762 (95.6)	146 (2.4)	119 (2.0)	68	24 (100.0)	0 (0.0)	0 (0.0)	0
Kept away from crowded places	2,162 (88.7)	176 (7.2)	99 (4.1)	55	5,649 (93.9)	190 (3.2)	178 (3.0)	78	23 (95.8)	1 (4.2)	0 (0.0)	0
Tried to avoid people who have the cold or flu-like symptoms	2,086 (85.8)	211 (8.7)	135 (5.6)	60	5,418 (90.4)	302 (5.0)	277 (4.6)	98	19 (86.4)	1 (4.6)	2 (9.1)	2
**Avoiding travel (%)**	**Y**	**N**	**NS**	**NA**	**Y**	**N**	**NS**	**NA**	**Y**	**N**	**NS**	**NA**
Reduced the amount of going to school/university/work	1,923 (89.2)	223 (10.3)	11 (0.5)	335	4,505 (87.5)	615 (11.9)	31 (0.6)	944	18 (90.0)	1 (5.0)	1 (5.0)	4
Reduced use of or change the way you use public transport	1,522 (85.9)	210 (11.9)	40 (2.3)	720	3,981 (91.2)	299 (6.9)	87 (2.0)	1,728	18 (90.0)	2 (10.0)	0 (0.0)	4
Reduced the amount you go to shops	1,808 (74.4)	480 (19.8)	142 (5.8)	62	5,060 (84.2)	671 (11.2)	278 (4.6)	86	20 (83.3)	3 (12.5)	1 (4.2)	0
Other behaviors not mentioned (%)	644 (26.8)	1,560 (64.9)	199 (8.3)	89	1,445 (25.1)	3,758 (65.2)	561 (9.7)	331	5 (22.7)	17 (77.3)	0 (0.0)	2

Y = Yes; N = No; NS = Not sure; NA = Not applicable. For full questionnaire and wording, see Supplement I.
Response percentages may not add up to 100% due to rounding

Table n: Individual implementation of protective measures in response to COVID-19 pandemic in the Netherlands, by age.

	≤20 years				21-40 years				41-60 years				> 60 years			
No.	675				4,034				2,836				1,066			
**Personal protective behaviors (%)**	**Y**	**N**	**NS**	**NA**	**Y**	**N**	**NS**	**NA**	**Y**	**N**	**NS**	**NA**	**Y**	**N**	**NS**	**NA**
Cleaned or disinfected things you might touch more often than usual	229 (34.5)	365 (55.0)	70 (10.5)	11	1,631 (40.7)	2,063 (51.5)	314 (7.8)	26	1,599 (56.6)	1,000 (35.4)	225 (8.0)	12	624 (59.3)	325 (30.9)	104 (9.9)	13
Carried sanitizing hand gel with you when out and about	180 (27.4)	460 (69.9)	18 (2.7)	17	1,101 (27.7)	2,788 (70.2)	80 (2.0)	65	809 (29.1)	1,919 (69.0)	55 (2.0)	53	295 (28.7)	704 (68.6)	28 (2.7)	39
Used sanitizing hand gel to clean your hands, more often than usual	383 (57.5)	268 (40.2)	15 (2.3)	9	2,135 (53.6)	1,773 (44.5)	77 (1.9)	49	1,552 (55.6)	1,175 (42.1)	63 (2.3)	46	611 (59.5)	385 (37.5)	31 (3.0)	39
Reduced the amount you touch your eyes, nose and/or mouth	299 (44.4)	223 (33.1)	152 (22.6)	1	1,909 (47.4)	1,268 (31.5)	851 (21.1)	6	1,479 (52.4)	730 (25.8)	616 (21.8)	11	521 (49.1)	270 (25.5)	270 (25.5)	5
Followed a healthy diet or took vitamin supplements	281 (42.6)	329 (49.9)	49 (7.4)	16	2,019 (51.3)	1,708 (43.4)	209 (5.3)	98	1,627 (59.3)	1,038 (37.9)	77 (2.8)	94	621 (61.5)	354 (35.1)	35 (3.5)	56
Usually carried tissues with you when out and about	209 (31.9)	427 (65.2)	19 (2.9)	20	1,544 (39.2)	2,306 (58.6)	87 (2.2)	97	1,774 (64.3)	936 (33.9)	48 (1.7)	78	872 (83.4)	164 (15.7)	9 (0.9)	21
Usually used tissues when sneezing or coughing	266 (44.3)	312 (51.9)	23 (3.8)	74	1,699 (50.3)	1,551 (45.9)	127 (3.8)	657	1,775 (74.5)	548 (23.0)	59 (2.5)	454	802 (83.1)	140 (14.5)	23 (2.4)	101
Washed your hands with soap and water more often than usual	629 (93.2)	39 (5.8)	7 (1.0)	0	3,797 (94.2)	208 (5.2)	27 (0.7)	2	2,720 (96.0)	93 (3.3)	19 (0.7)	4	1,030 (96.8)	31 (2.9)	3 (0.3)	2
Kept child(ren) at home before any mandates were put in place	25 (69.4)	9 (25.0)	2 (5.6)	639	473 (56.4)	349 (41.6)	17 (2.0)	3,195	588 (49.9)	579 (49.1)	12 (1.0)	1,657	65 (70.7)	20 (21.7)	7 (7.6)	974
**Limiting interactions with people (%)**	**Y**	**N**	**NS**	**NA**	**Y**	**N**	**NS**	**NA**	**Y**	**N**	**NS**	**NA**	**Y**	**N**	**NS**	**NA**
Cancelled or postponed a social event	571 (85.9)	52 (7.8)	42 (6.3)	10	3,720 (93.4)	162 (4.1)	101 (2.5)	51	2,739 (97.7)	37 (1.3)	27 (1.0)	33	1,012 (97.6)	17 (1.6)	8 (0.8)	29
Kept away from crowded places	552 (83.5)	65 (9.8)	44 (6.7)	14	3,576 (90.1)	223 (5.6)	169 (4.3)	66	2,691 (96.2)	55 (2.0)	52 (1.9)	38	1,015 (96.6)	24 (2.3)	12 (1.1)	1
Tried to avoid people who have the cold or flu-like symptoms	521 (79.9)	68 (10.4)	63 (9.7)	23	3,447 (87.0)	297 (7.5)	217 (5.5)	73	2,570 (92.2)	113 (4.1)	104 (3.7)	49	985 (93.7)	36 (3.4)	30 (2.9)	15
**Avoiding travel (%)**	**Y**	**N**	**NS**	**NA**	**Y**	**N**	**NS**	**NA**	**Y**	**N**	**NS**	**NA**	**Y**	**N**	**NS**	**NA**
Reduced the amount of going to school/university/work	639 (95.8)	28 (4.2)	0 (0.0)	8	3,438 (91.4)	304 (8.1)	20 (0.5)	272	1,987 (81.6)	430 (17.67)	17 (0.7)	402	382 (82.2)	77 (16.6)	6 (1.3)	601
Reduced use of or change the way you use public transport	490 (82.5)	81 (13.6)	23 (3.9)	81	2,854 (88.3)	298 (9.2)	81 (2.5)	801	1,619 (94.0)	88 (5.1)	16 (0.9)	1,113	558 (91.6)	44 (7.2)	7 (1.2)	457
Reduced the amount you go to shops	486 (75.2)	115 (17.8)	45 (7.0)	29	3,155 (79.7)	566 (14.3)	238 (6.0)	75	2,369 (84.3)	343 (12.2)	97 (3.5)	27	878 (83.7)	130 (12.4)	41 (3.9)	17
Other behaviors not mentioned	139 (21.5)	415 (64.1)	93 (14.4)	2	985 (25.5)	2,481 (64.3)	391 (10.1)	177	707 (26.3)	1,784 (66.3)	201 (7.5)	144	263 (26.5)	655 (66.0)	75 (7.6)	73

Y = Yes; N = No; NS = Not sure; NA = Not applicable. For full questionnaire and wording, see Supplement I.
Response percentages may not add up to 100% due to rounding

Table o: Individual implementation of protective measures in response to COVID-19 pandemic in the Netherlands, by daily activity.

	Paid Job				Homemaker/ Unemployed				Student				Retired				Other			
No.	4,885				573				2,272				557				324			
**Personal protective behaviors (%)**	**Y**	**N**	**NS**	**NA**	**Y**	**N**	**NS**	**NA**	**Y**	**N**	**NS**	**NA**	**Y**	**N**	**NS**	**NA**	**Y**	**N**	**NS**	**NA**
Cleaned or disinfected things you might touch more often than usual	2,458 (50.6)	2,022 (41.6)	380 (7.8)	25	340 (59.3)	178 (31.1)	55 (9.6)	0	791 (35.1)	1,262 (56.1)	198 (8.8)	21	324 (59.3)	174 (31.9)	48 (8.8)	11	170 (53.3)	117 (36.7)	32 (10.0)	5
Carried sanitizing hand gel with you when out and about	1,380 (28.6)	3,346 (69.5)	92 (1.9)	67	171 (30.8)	373 (67.2)	11 (2.0)	18	572 (25.6)	1,602 (71.8)	58 (2.6)	40	151 (28.3)	366 (68.7)	16 (2.6)	24	111 (37.1)	184 (61.5)	4 (1.3)	25
Used sanitizing hand gel to clean your hands, more often than usual	2,722 (56.4)	2,016 (41.8)	86 (1.8)	61	292 (52.3)	245 (43.9)	21 (3.8)	15	1,193 (53.3)	989 (44.2)	58 (2.6)	32	311 (58.5)	206 (38.7)	15 (2.8)	25	163 (51.9)	145 (46.2)	6 (1.9)	10
Reduced the amount you touch your eyes, nose and/or mouth	2,425 (49.8)	1,398 (28.7)	1,050 (21.6)	12	307 (53.9)	135 (23.7)	128 (22.5)	3	1,021 (45.0)	744 (32.8)	503 (22.2)	4	279 (50.4)	136 (24.6)	139 (25.1)	3	176 (54.5)	78 (24.2)	69 (21.4)	1
Followed a healthy diet or took vitamin supplements	2,612 (55.1)	1,952 (41.2)	175 (3.7)	146	334 (61.1)	196 (35.8)	17 (3.1)	26	1,076 (48.3)	1,010 (45.3)	143 (6.4)	43	343 (64.8)	168 (31.8)	18 (3.4)	28	183 (60.4)	103 (34.0)	17 (5.6)	21
Usually carried tissues with you when out and about	2,622 (55.0)	2,060 (43.2)	87 (1.8)	116	372 (68.0)	165 (30.2)	10 (1.8)	26	739 (33.3)	1,426 (64.2)	57 (2.6)	50	465 (84.7)	80 (14.6)	4 (0.7)	8	201 (65.3)	102 (33.1)	5 (1.6)	16
Usually used tissues when sneezing or coughing	2,742 (66.2)	1,281 (30.9)	121 (2.9)	741	350 (72.2)	120 (24.7)	15 (3.1)	88	826 (43.3)	1,002 (52.5)	80 (4.2)	364	426 (82.9)	79 (15.4)	9 (1.8)	43	198 (72.3)	69 (25.2)	7 (2.6)	50
Washed your hands with soap and water more often than usual	4,658 (95.5)	192 (3.9)	29 (0.6)	6	538 (94.1)	30 (5.2)	4 (0.7)	1	2,133 (93.9)	120 (5.3)	19 (0.8)	0	537 (96.6)	17 (3.1)	2 (0.4)	1	310 (95.7)	12 (3.7)	2 (0.6)	0
Kept child(ren) at home before any mandates were put in place	882 (51.1)	822 (47.7)	21 (1.2)	3,160	142 (62.6)	81 (35.7)	4 (1.8)	346	55 (68.8)	19 (23.8)	6 (7.5)	2,192	34 (70.8)	9 (18.8)	5 (10.4)	509	38 (57.6)	26 (39.4)	2 (3.0)	258
**Limiting interactions with people (%)**	**Y**	**N**	**NS**	**NA**	**Y**	**N**	**NS**	**NA**	**Y**	**N**	**NS**	**NA**	**Y**	**N**	**NS**	**NA**	**Y**	**N**	**NS**	**NA**
Cancelled or postponed a social event	4,656 (96.3)	115 (2.4)	64 (1.3)	50	540 (97.5)	7 (1.3)	7 (1.3)	19	2,015 (89.8)	134 (6.0)	95 (4.2)	28	531 (98.3)	6 (1.1)	3 (0.6)	17	300 (95.2)	6 (1.9)	9 (2.9)	9
Kept away from crowded places	4,526 (93.8)	176 (3.7)	121 (2.5)	62	543 (97.0)	7 (1.3)	10 (1.8)	13	1,928 (86.4)	170 (7.6)	134 (6.0)	40	537 (98.0)	6 (1.1)	5 (0.9)	9	300 (95.2)	8 (2.5)	7 (2.2)	9
Tried to avoid people who have the cold or flu-like symptoms	4,346 (90.5)	267 (5.6)	191 (4.0)	81	527 (93.4)	16 (2.8)	21 (3.7)	9	1,834 (82.5)	209 (9.4)	179 (8.1)	50	520 (95.1)	13 (2.4)	14 (2.6)	10	296 (94.3)	9 (2.9)	9 (2.9)	10
**Avoiding travel (%)**	**Y**	**N**	**NS**	**NA**	**Y**	**N**	**NS**	**NA**	**Y**	**N**	**NS**	**NA**	**Y**	**N**	**NS**	**NA**	**Y**	**N**	**NS**	**NA**
Reduced the amount of going to school/university/work	3,684 (82.7)	744 (16.7)	26 (0.6)	431	298 (90.0)	29 (8.8)	4 (1.2)	242	2,189 (97.9)	44 (2.0)	3 (0.1)	36	103 (91.2)	6 (5.3)	4 (3.5)	444	172 (88.7)	16 (8.3)	6 (3.1)	130
Reduced use of or change the way you use public transport	3,007 (93.1)	194 (6.0)	30 (0.9)	1,654	307 (92.8)	19 (5.7)	5 (1.5)	242	1,744 (83.3)	267 (12.8)	83 (4.0)	178	270 (90.9)	22 (7.4)	5 (1.7)	260	193 (93.7)	9 (4.4)	4 (1.9)	118
Reduced the amount you go to shops	4,009 (83.1)	616 (12.8)	202 (4.2)	58	487 (85.4)	57 (10.0)	26 (4.6)	3	1,652 (75.0)	381 (17.3)	169 (7.7)	70	462 (84.5)	70 (12.8)	15 (2.7)	10	278 (87.7)	30 (9.5)	9 (2.8)	7
Other behaviors not mentioned	1,163 (25.1)	3,109 (67.1)	364 (7.9)	249	146 (26.8)	347 (63.8)	51 (9.4)	29	529 (24.2)	1,375 (62.9)	281 (12.9)	87	128 (24.6)	355 (68.3)	37 (7.1)	37	128 (42.1)	149 (49.0)	27 (8.9)	20

Y = Yes; N = No; NS = Not sure; NA = Not applicable. For full questionnaire and wording, see Supplement I.
Response percentages may not add up to 100% due to rounding

Table p: Individual implementation of protective measures in response to COVID-19 pandemic in the Netherlands, by household composition.

	Single				Parent(s) with child(ren)				Living with partner				Shared flat				Other			
No.	1,391				2,740				2,339				1,558				583			
**Personal protective behaviors (%)**	**Y**	**N**	**NS**	**NA**	**Y**	**N**	**NS**	**NA**	**Y**	**N**	**NS**	**NA**	**Y**	**N**	**NS**	**NA**	**Y**	**N**	**NS**	**NA**
Cleaned or disinfected things you might touch more often than usual	555 (40.4)	701 (51.0)	118 (8.6)	17	1,525 (55.8)	987 (36.1)	220 (8.1)	8	1,218 (52.4)	919 (39.5)	189 (8.1)	13	558 (36.0)	857 (55.3)	134 (8.7)	9	227 (40.0)	289 (50.9)	52 (9.2)	15
Carried sanitizing hand gel with you when out and about	365 (27.0)	960 (71.1)	26 (1.9)	40	768 (28.6)	1,862 (69.2)	60 (2.2)	50	687 (30.0)	1,562 (68.2)	41 (1.8)	49	369 (24.1)	1,120 (73.1)	43 (2.8)	26	196 (34.2)	367 (63.9)	11 (1.9)	9
Used sanitizing hand gel to clean your hands, more often than usual	666 (48.9)	665 (48.8)	32 (2.4)	28	1,553 (57.4)	1,094 (40.4)	59 (2.2)	34	1,297 (56.7)	946 (41.4)	45 (2.0)	51	831 (54.2)	666 (43.4)	36 (2.4)	25	334 (57.8)	230 (39.8)	14 (2.4)	5
Reduced the amount you touch your eyes, nose and/or mouth	683 (49.4)	415 (30.0)	286 (20.7)	7	1,405 (51.4)	755 (27.6)	574 (21.0)	6	1,163 (49.9)	615 (26.4)	553 (23.7)	8	695 (44.6)	518 (33.3)	344 (22.1)	1	262 (45.0)	188 (32.3)	132 (22.7)	1
Followed a healthy diet or took vitamin supplements	776 (57.3)	516 (38.1)	63 (4.7)	36	1,493 (56.3)	1,083 (40.8)	78 (2.9)	86	1,286 (57.3)	857 (38.2)	102 (4.5)	94	762 (49.7)	675 (44.1)	95 (6.2)	26	231 (41.2)	298 (53.1)	32 (5.7)	22
Usually carried tissues with you when out and about	755 (55.8)	568 (42.0)	29 (2.1)	39	1,553 (58.3)	1,064 (40.0)	45 (1.7)	78	1,407 (61.6)	840 (36.8)	39 (1.7)	53	478 (31.3)	1,014 (66.4)	35 (2.3)	31	206 (36.3)	347 (61.1)	15 (2.6)	15
Usually used tissues when sneezing or coughing	707 (62.2)	393 (34. 6)	37 (3.3)	254	1,636 (69.9)	642 (27.4)	64 (2.7)	398	1,393 (68.9)	563 (27.8)	66 (3.3)	317	571 (43.1)	707 (53.4)	46 (3.5)	234	235 (47.0)	246 (49.2)	19 (3.8)	83
Washed your hands with soap and water more often than usual	1,295 (93.1)	83 (6.0)	13 (0.9)	0	2,631 (96.1)	95 (3.5)	12 (0.4)	2	2,242 (96.1)	78 (3.3)	13 (0.6)	6	1,481 (95.1)	65 (4.2)	12 (0.8)	0	527 (90.4)	50 (8.6)	6 (1.0)	0
Kept child(ren) at home before any mandates were put in place	38 (70.4)	12 (22.2)	4 (7.4)	1,337	974 (51.4)	901 (47.5)	21 (1.1)	844	89 (73.6)	25 (20.7)	7 (5.8)	2,218	27 (60.0)	14 (31.1)	4 (8.9)	1,513	23 (76.7)	5 (16.7)	2 (6.7)	553
**Limiting interactions with people (%)**	**Y**	**N**	**NS**	**NA**	**Y**	**N**	**NS**	**NA**	**Y**	**N**	**NS**	**NA**	**Y**	**N**	**NS**	**NA**	**Y**	**N**	**NS**	**NA**
Cancelled or postponed a social event	1,276 (93.8)	55 (4.0)	30 (2.2)	30	2,640 (97.5)	41 (1.5)	28 (1.0)	31	2,222 (96.4)	53 (2.3)	29 (1.3)	35	1,410 (91.1)	72 (4.7)	65 (4.2)	11	494 (87.1)	47 (8.3)	26 (4.6)	16
Kept away from crowded places	1,246 (91.2)	74 (5.4)	46 (3.4)	25	2,597 (96.1)	63 (2.3)	43 (1.6)	37	2,203 (95.3)	55 (2.4)	53 (2.3)	28	1,311 (85.5)	123 (8.0)	100 (6.5)	24	477 (84.6)	52 (9.2)	35 (6.2)	19
Tried to avoid people who have the cold or flu-like symptoms	1,205 (88.9)	91 (6.7)	60 (4.4)	35	2,461 (91.3)	124 (4.6)	111 (4.1)	44	2,137 (92.9)	88 (3.8)	76 (3.3)	38	1,257 (82.2)	156 (10.2)	116 (7.6)	29	463 (81.4)	55 (9.7)	51 (9.0)	14
**Avoiding travel (%)**	**Y**	**N**	**NS**	**NA**	**Y**	**N**	**NS**	**NA**	**Y**	**N**	**NS**	**NA**	**Y**	**N**	**NS**	**NA**	**Y**	**N**	**NS**	**NA**
Reduced the amount of going to school/university/work	999 (88.3)	125 (11.0)	8 (0.7)	259	2,018 (83.4)	389 (16.1)	12 (0.5)	321	1,468 (85.4)	237 (13.8)	14 (0.8)	620	1,452 (96.2)	51 (3.4)	7 (0.5)	48	509 (92.9)	37 (6.8)	2 (0.4)	35
Reduced use of or change the way you use public transport	920 (88.0)	95 (9.1)	30 (2.9)	346	1,624 (95.4)	69 (4.1)	9 (0.5)	1,038	1,391 (91.9)	104 (6.9)	19 (1.3)	825	1,164 (82.0)	197 (13.9)	58 (4.1)	139	422 (88.1)	46 (9.6)	11 (2.3)	104
Reduced the amount you go to shops	1,080 (79.1)	210 (15.4)	76 (5.6)	25	2,315 (85.5)	312 (11.5)	82 (3.0)	31	1,935 (83.9)	268 (11.6)	104 (4.5)	32	1,163 (76.0)	257 (16.8)	111 (7.3)	27	395 (71.8)	107 (19.5)	48 (8.7)	33
Other behaviors not mentioned	329 (24.8)	857 (64.6)	141 (10.6)	64	701 (26.8)	1,728 (66.0)	188 (7.2)	123	570 (25.9)	1,439 (65.3)	194 (8.8)	136	350 (23.4)	967 (64.7)	177 (11.9)	64	144 (26.3)	344 (62.8)	60 (11.0)	35

Y = Yes; N = No; NS = Not sure; NA = Not applicable. For full questionnaire and wording, see Supplement I.
Response percentages may not add up to 100% due to rounding

Table q: Individual implementation of protective measures in response to COVID-19 pandemic in the Netherlands, by highest educational qualification.

	Primary/Secondary				Tertiary vocational				Tertiary academic			
No.	635				1,991				5,985			
**Personal protective behaviors (%)**	**Y**	**N**	**NS**	**NA**	**Y**	**N**	**NS**	**NA**	**Y**	**N**	**NS**	**NA**
Cleaned or disinfected things you might touch more often than usual	370 (59.1)	200 (32.0)	56 (9.0)	9	1,127 (57.2)	677 (34.4)	167 (8.5)	20	2,586 (43.5)	2,876 (48.3)	490 (8.2)	33
Carried sanitizing hand gel with you when out and about	226 (37.1)	369 (60.5)	15 (2.5)	25	622 (32.2)	1,264 (65.5)	43 (2.2)	62	1,537 (26.1)	4,238 (71.9)	123 (2.1)	87
Used sanitizing hand gel to clean your hands, more often than usual	405 (64.7)	203 (32.4)	18 (2.9)	9	1,205 (61.6)	712 (36.4)	38 (1.9)	36	3,071 (52.2)	2,686 (45.6)	130 (2.2)	98
Reduced the amount you touch your eyes, nose and/or mouth	330 (52.3)	174 (27.6)	127 (20.1)	4	1,025 (51.6)	535 (26.9)	428 (21.5)	3	2,853 (47.8)	1,782 (29.9)	1,334 (22.4)	16
Followed a healthy diet or took vitamin supplements	329 (53.6)	264 (43.0)	21 (3.4)	21	1,023 (53.5)	809 (42.3)	81 (4.2)	78	3,196 (54.9)	2,356 (40.5)	268 (4.6)	165
Usually carried tissues with you when out and about	396 (65.1)	201 (33.1)	11 (1.8)	27	1,150 (59.0)	764 (39.2)	36 (1.9)	41	2,853 (48.9)	2,868 (49.1)	116 (2.0)	148
Usually used tissues when sneezing or coughing	405 (70.2)	156 (27.0)	16 (2.8)	58	1,162 (67.1)	513 (29.6)	57 (3.3)	259	2,975 (59.3)	1,882 (37.5)	159 (3.2)	969
Washed your hands with soap and water more often than usual	608 (95.9)	19 (3.0)	7 (1.1)	1	1,879 (94.7)	95 (4.8)	11 (0.6)	6	5,689 (95.1)	257 (4.3)	38 (0.6)	1
Kept child(ren) at home before any mandates were put in place	73 (57.5)	52 (40.9)	2 (1.6)	508	331 (57.8)	231 (40.3)	11 (1.9)	1,418	747 (51.7)	674 (46.6)	25 (1.7)	4,539
**Limiting interactions with people (%)**	**Y**	**N**	**NS**	**NA**	**Y**	**N**	**NS**	**NA**	**Y**	**N**	**NS**	**NA**
Cancelled or postponed a social event	576 (94.3)	26 (4.3)	9 (1.5)	24	1,849 (94.8)	54 (2.8)	47 (2.4)	41	5,617 (94.8)	188 (3.2)	122 (2.1)	58
Kept away from crowded places	581 (92.5)	23 (3.7)	24 (3.8)	7	1,811 (92.6)	82 (4.2)	62 (3.2)	36	5,442 (92.3)	262 (4.4)	191 (3.2)	90
Tried to avoid people who have the cold or flu-like symptoms	540 (87.5)	32 (5.2)	45 (7.3)	18	1,754 (89.7)	103 (5.3)	99 (5.1)	35	5,229 (89.0)	379 (6.5)	270 (4.6)	107
**Avoiding travel (%)**	**Y**	**N**	**NS**	**NA**	**Y**	**N**	**NS**	**NA**	**Y**	**N**	**NS**	**NA**
Reduced the amount of going to school/university/work	334 (75.4)	99 (22.4)	10 (2.3)	192	1,191 (77.0)	343 (22.2)	13 (0.8)	444	4,921 (92.2)	397 (7.4)	20 (0.4)	647
Reduced use of or change the way you use public transport	336 (85.7)	43 (11.0)	13 (3.3)	243	1,113 (89.7)	108 (8.7)	20 (1.6)	750	4,072 (90.0)	360 (8.0)	94 (2.1)	1,459
Reduced the amount you go to shops	518 (82.6)	80 (12.8)	29 (4.6)	8	1,624 (83.4)	233 (12.0)	91 (4.7)	43	4,746 (80.6)	841 (14.3)	301 (5.1)	97
Other behaviors not mentioned	148 (25.3)	370 (63.4)	66 (11.3)	51	472 (25.1)	1,238 (65.8)	173 (9.2)	108	1,474 (25.8)	3,727 (65.1)	521 (9.1)	263

Y = Yes; N = No; NS = Not sure; NA = Not applicable. For full questionnaire and wording, see Supplement I.
Response percentages may not add up to 100% due to rounding.

Table r: Individual implementation of protective measures in response to COVID-19 pandemic in the Netherlands, by healthcare provider or (bio-)medical student, and by chronic illness or being in poor medical condition.

	Healthcare provider or (bio-)medical student								Chronic medical condition							
	Yes				No				Yes				No			
No.	1,572				7,039				7,083				1,528			
**Personal protective behaviors (%)**	**Y**	**N**	**NS**	**NA**	**Y**	**N**	**NS**	**NA**	**Y**	**N**	**NS**	**NA**	**Y**	**N**	**NS**	**NA**
Cleaned or disinfected things you might touch more often than usual	782 (50.0)	664 (42.5)	118 (7.5)	8	3,301 (47.3)	3,089 (44.2)	595 (8.5)	54	879 (58.0)	525 (34.7)	111 (7.3)	13	3,204 (45.6)	3,228 (45.9)	602 (8.6)	49
Carried sanitizing hand gel with you when out and about	473 (30.6)	1,044 (67.4)	31 (2.0)	24	1,912 (27.8)	4,827 (70.1)	150 (2.2)	150	493 (33.3)	959 (64.8.9)	27 (1.8)	49	1,892 (33.3)	4,912 (64.8)	154 (1.8)	125
Used sanitizing hand gel to clean your hands, more often than usual	914 (58.7)	610 (39.2)	32 (2.1)	16	3,767 (54.5)	2,991 (43.3)	154 (2.2)	127	868 (57.9)	599 (40.0)	33 (2.2)	28	3,813 (54.7)	3,002 (43.1)	153 (2.2)	115
Reduced the amount you touch your eyes, nose and/or mouth	761 (48.5)	462 (29.5)	346 (22.1)	3	3,447 (49.1)	2,029 (28.9)	1,543 (22.0)	20	813 (53.4)	386 (25.4)	323 (21.2)	6	3,395 (48.1)	2,105 (29.8)	1,566 (22.2)	17
Followed a healthy diet or took vitamin supplements	758 (49.6)	701 (45.9)	70 (4.6)	43	3,790 (55.6)	2,728 (40.0)	300 (4.4)	221	871 (58.9)	549 (37.1)	60 (4.1)	48	3,677 (53.6)	2,880 (41.9)	310 (4.5)	216
Usually carried tissues with you when out and about	728 (47.6)	767 (50.1)	36 (2.4)	41	3,671 (53.5)	3,066 (44.7)	127 (1.9)	175	993 (66.9)	467 (31.5)	25 (1.7)	43	3,406 (49.3)	3,366 (48.7)	138 (2.0)	173
Usually used tissues when sneezing or coughing	756 (59.0)	486 (37.9)	40 (3.1)	290	3,786 (62.7)	2.065 (34.2)	192 (3.2)	996	976 (73.0)	319 (23.9)	42 (3.1)	191	3,566 (59.6)	2,232 (37.3)	190 (3.2)	1,095
Washed your hands with soap and water more often than usual	1,479 (94.2)	80 (5.1)	11 (0.7)	2	6,697 (95.2)	291 (4.1)	45 (0.6)	6	1,462 (95.7)	58 (3.8)	8 (0.5)	0	6,714 (94.9)	313 (4.4)	48 (0.7)	8
Kept child(ren) at home before any mandates were put in place	178 (48.9)	180 (49.5)	6 (1.7)	1,208	973 (54.6)	777 (43.6)	32 (1.8)	5,257	209 (57.7)	143 (39.5)	10 (2.8)	1,166	942 (52.8)	814 (45.6)	28 (1.6)	5,299
**Limiting interactions with people (%)**	**Y**	**N**	**NS**	**NA**	**Y**	**N**	**NS**	**NA**	**Y**	**N**	**NS**	**NA**	**Y**	**N**	**NS**	**NA**
Cancelled or postponed a social event	1,474 (94.3)	52 (3.3)	38 (2.4)	8	6,568 (94.9)	216 (3.1)	140 (2.0)	115	1,443 (97.1)	29 (2.0)	14 (1.0)	42	6,599 (94.2)	239 (3.4)	164 (2.3)	81
Kept away from crowded places	1,429 (92.4)	67 (4.3)	51 (3.3)	25	6,405 (92.4)	300 (4.3)	226 (3.3)	108	1,441 (95.5)	40 (2.7)	28 (1.9)	19	6,393 (91.7)	327 (4.7)	249 (3.6)	114
Tried to avoid people who have the cold or flu-like symptoms	1,352 (87.9)	100 (6.5)	86 (5.6)	34	6,171 (89.3)	414 (6.0)	328 (4.7)	126	1,386 (92.4)	62 (4.1)	52 (3.5)	28	6,137 (88.3)	452 (6.5)	362 (5.2)	132
**Avoiding travel (%)**	**Y**	**N**	**NS**	**NA**	**Y**	**N**	**NS**	**NA**	**Y**	**N**	**NS**	**NA**	**Y**	**N**	**NS**	**NA**
Reduced the amount of going to school/university/work	1,061 (77.5)	303 (22.1)	6 (0.4)	202	5,385 (90.4)	536 (9.0)	37 (0.6)	1,081	949 (85.3)	153 (13.8)	10 (0.9)	416	5,497 (88.4)	686 (11.0)	33 (0.5)	867
Reduced use of or change the way you use public transport	1,014 (87.0)	124 (10.6)	27 (2.3)	407	4,507 (90.3)	387 (7.8)	100 (2.0)	2,045	896 (91.8)	68 (7.0)	12 (1.2)	552	4,625 (89.2)	443 (8.6)	115 (2.2)	1,900
Reduced the amount you go to shops	1,271 (82.3)	213 (13.8)	60 (3.9)	28	5,617 (81.2)	941 (13.6)	361 (5.2)	120	1,296 (86.2)	156 (10.4)	51 (3.4)	25	5,592 (80.3)	998 (14.3)	370 (5.3)	123

Y = Yes; N = No; NS = Not sure; NA = Not applicable. For full questionnaire and wording, see Supplement I.
Response percentages may not add up to 100% due to rounding
