# Supplementary material for: Public perspectives on protective measures during the COVID-19 pandemic in the Netherlands, Germany and Italy: A survey study
Source: PLoS One. 2020 Aug 5;15(8):e0236917. doi: 10.1371/journal.pone.0236917 (PMC7406072; doi:10.1371/journal.pone.0236917)

S4 Appendix 4.

Fig 1a: Change over time in the proportion of positive answers ("Probably true"), excluding responses indicating the question was not applicable to their situation. Proportions were modeled for each item and for each country separately using generalized additive models with time as the independent variable, using a shrinkage version of cubic splines with three knots.

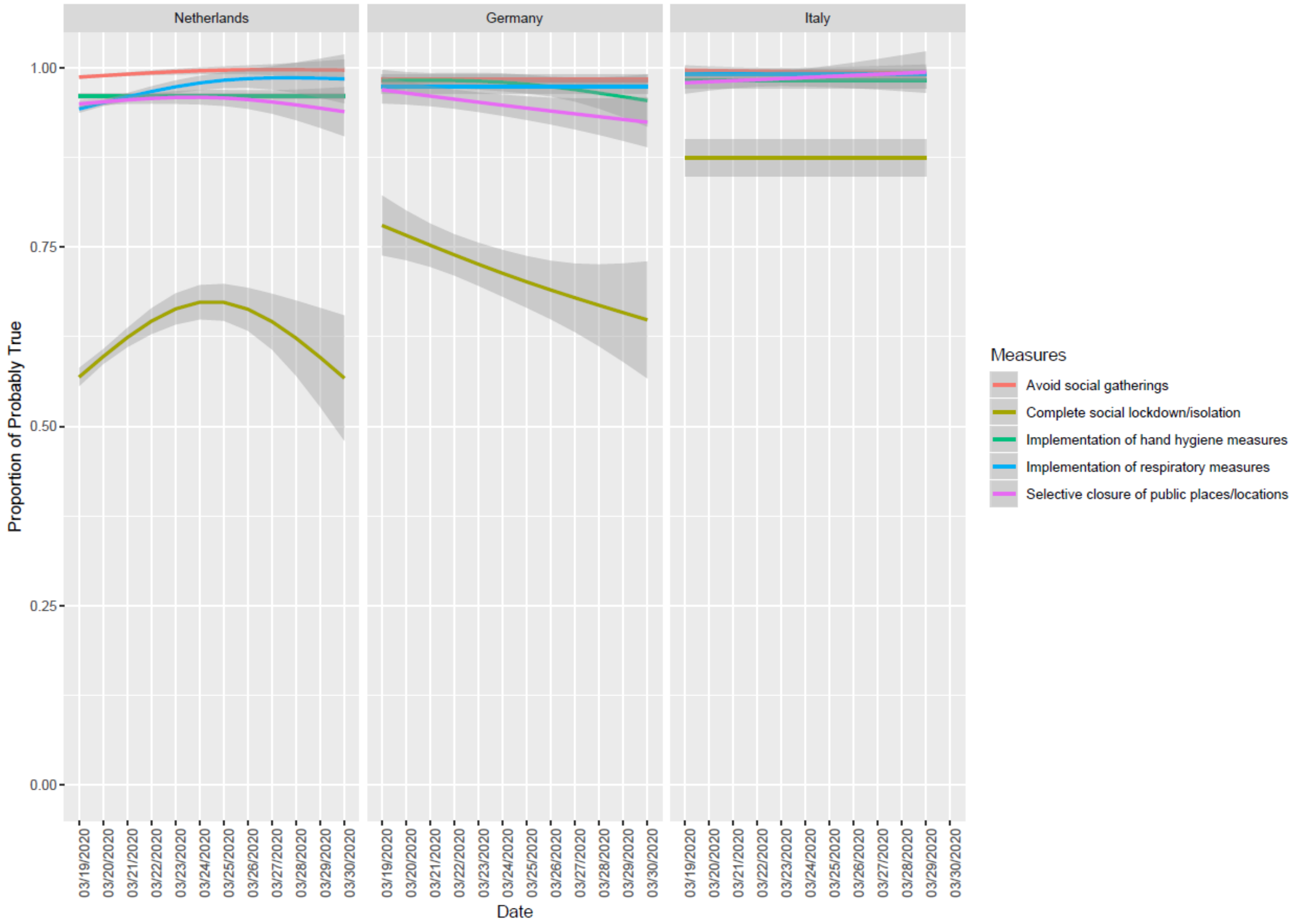

Fig 1b: Change over time in the proportion of positive answers (“Yes”) out of all responses, excluding responses indicating the question was not applicable to their situation. Proportions were modeled for each item and for each country separately using generalized additive models with time as independent variable, using a shrinkage version of cubic splines with three knots.

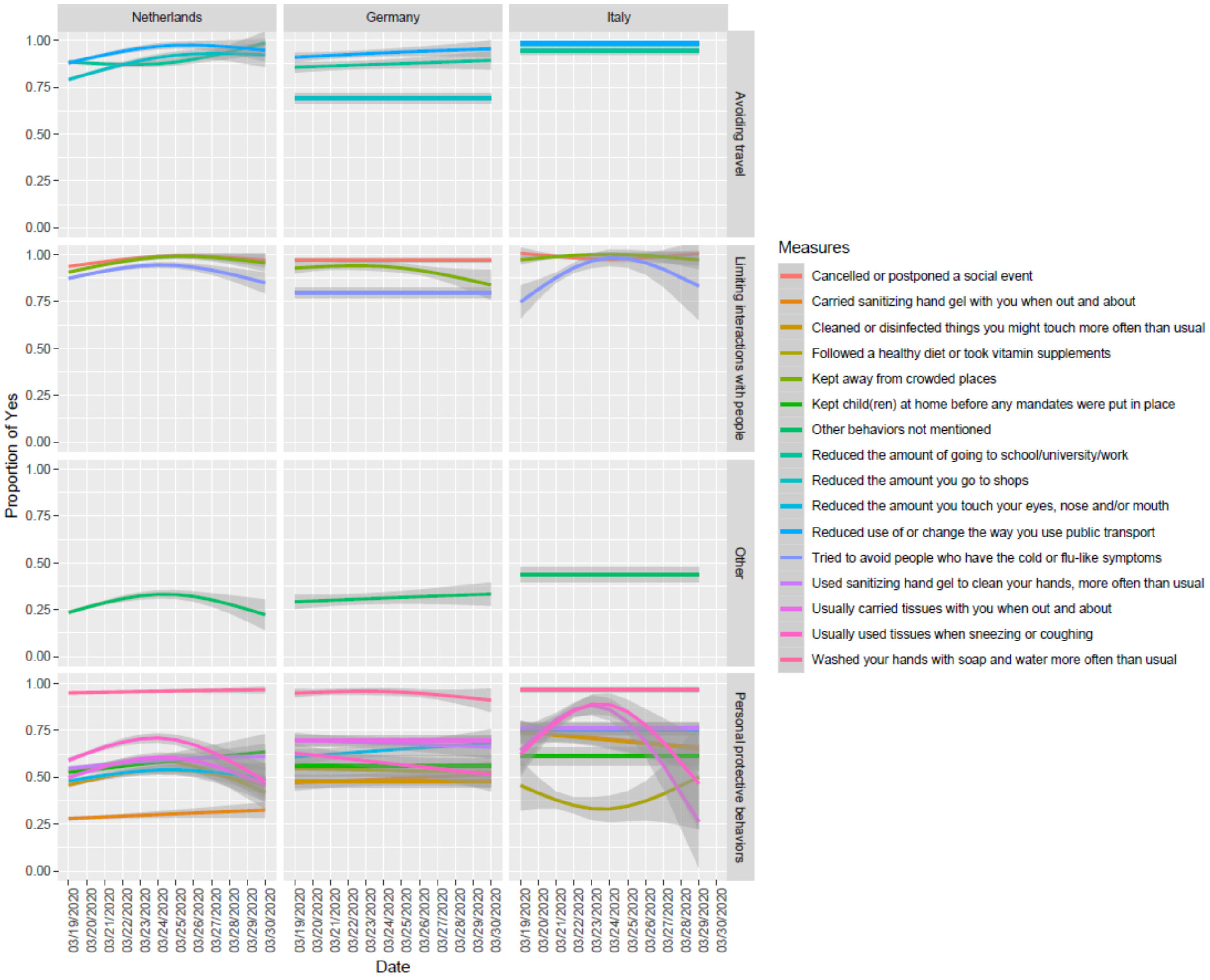

Fig 2a: Differences in percentages of item responses pertaining to beliefs in the effectiveness of protective measures between the weeklong extension and the primary data collection period among respondents living in the Netherlands. Note: Percentages are rounded and may not add up to zero.

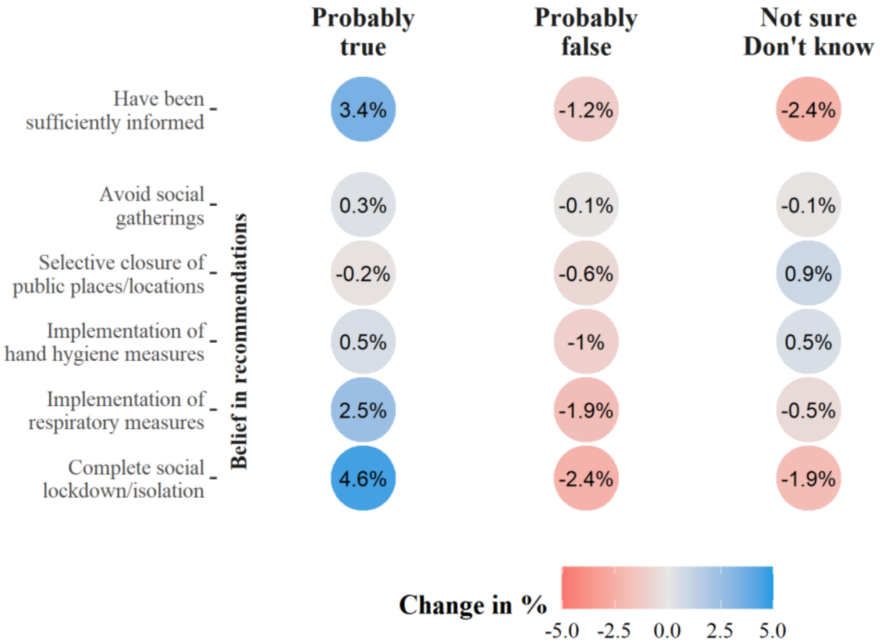

Fig 2b. Differences in percentages of item responses pertaining to the implementation of protective measures between the weeklong extension and the primary data collection period among respondents living in the Netherlands. Note: Percentages are rounded and may not add up to zero.

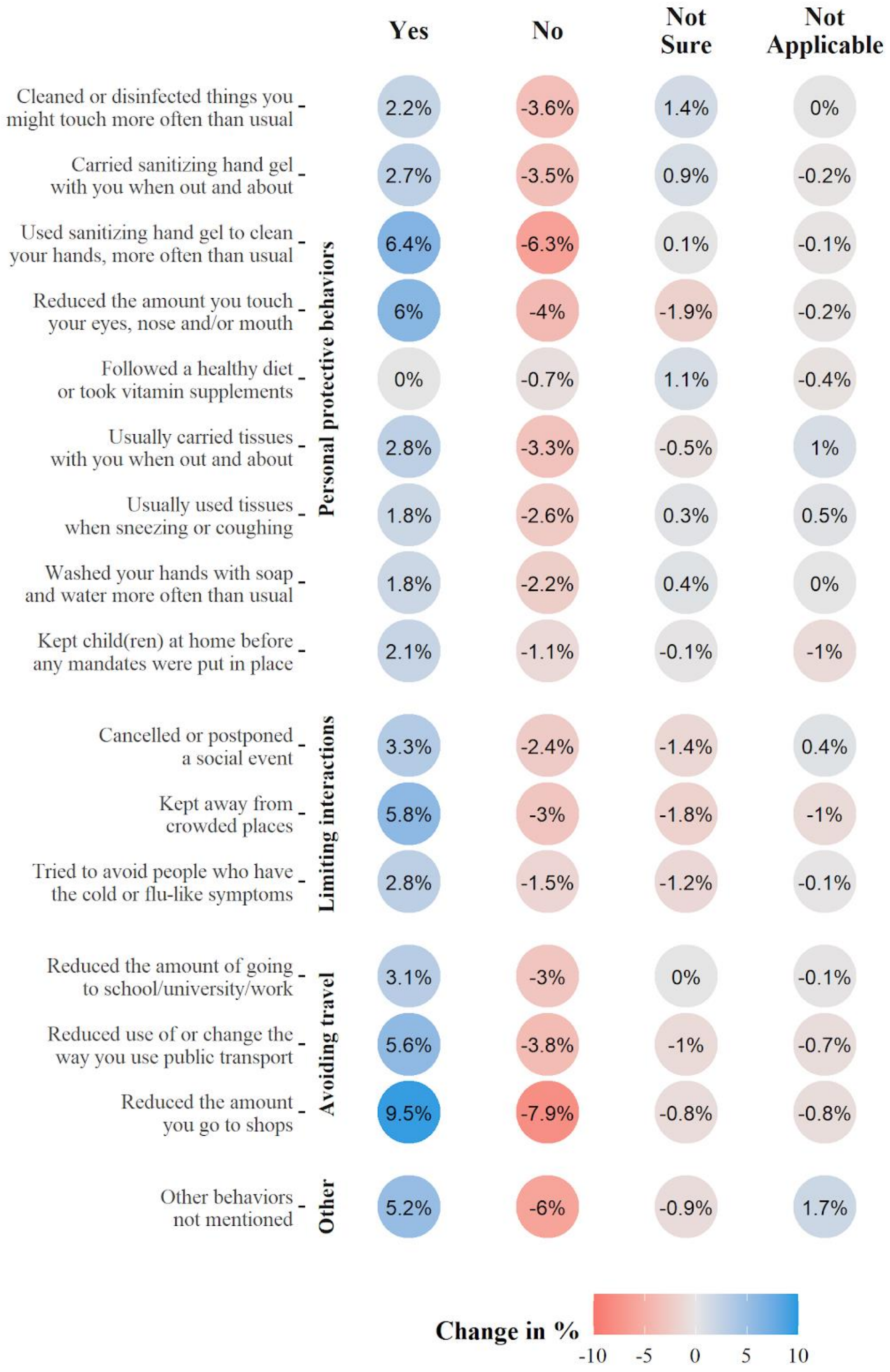

Supplement: S4 Appendix — (PDF) [file pone.0236917.s005.pdf]
